# The Duality of Collagens in Metastases of Solid Tumors

**DOI:** 10.3390/ijms26199745

**Published:** 2025-10-07

**Authors:** Michelle Carnazza, Danielle Quaranto, Nicole DeSouza, Xiu-Min Li, Raj K. Tiwari, Julie S. Di Martino, Jan Geliebter

**Affiliations:** 1 R & D Division, General Nutraceutical Technology, LLC, Briarcliff Manor, NY 10510, USA; michelle.carnazza@gnt-us.com; 2 Department of Pathology, Microbiology & Immunology, New York Medical College, Valhalla, NY 10595, USA; dquarant@student.touro.edu (D.Q.); ndesouza@student.touro.edu (N.D.); xiumin_li@nymc.edu (X.-M.L.); raj_tiwari@nymc.edu (R.K.T.); 3 Department of Otolaryngology, New York Medical College, Valhalla, NY 10595, USA; 4 Department of Dermatology, New York Medical College, Valhalla, NY 10595, USA; 5 Department of Cell Biology & Anatomy, New York Medical College, Valhalla, NY 10595, USA; jdimarti@nymc.edu

**Keywords:** cancer metastasis, tumor microenvironment, extracellular matrix, collagen, tumor invasion

## Abstract

Metastases are responsible for the majority of cancer-related deaths and remain one of the most complex and therapeutically challenging hallmarks of cancer. The metastatic cascade involves a multistep process by which cancer cells invade local tissue, enter and survive in circulation, extravasate, and ultimately colonize distant organs. Increasingly, the tumor microenvironment (TME), particularly the extracellular matrix (ECM), has emerged as a central regulator of these steps. Far from being a passive scaffold, the ECM actively influences cancer progression through its biochemical signals, structural properties, and dynamic remodeling. Among ECM components, collagens play a particularly pivotal role by mediating tumor cell adhesion, migration, invasion, survival, immune evasion, and therapeutic resistance. This narrative review synthesizes current knowledge of the dual roles of collagen in the metastatic process, with a focus on the cellular and molecular mechanisms. We highlight how altered ECM architecture and signaling contribute to metastatic niche formation and explore the potential of targeting ECM components as a strategy to enhance cancer therapy and improve patient outcomes.

## 1. Introduction

Metastases are responsible for ~90% of cancer-related lethal outcomes, and typically, symptoms of metastasis do not occur until the secondary organ function is compromised [1]. Hence, an important area of cancer study is the prevention of invasion and metastasis. Thus, the activation of invasion and metastasis is a hallmark of cancer distinguishes benign from malignant tumors [1,2]. The stages of metastasis include angiogenesis, induction of the epithelial–mesenchymal transition, migration, invasion of surrounding tissue, penetration of vascular channels, release into circulation as single cells or large clumps, survival, arrest at a secondary site of tumor growth, and colonization, whereby a secondary tumor is formed. As the tumor cell surroundings play crucial roles in the metastatic process, research focus has shifted to include the tumor microenvironment (TME), rather than solely the tumor itself, at both the primary tumor site and metastatic niche. In the context of cancer, the TME consists of immune and stromal cells and the extracellular matrix (ECM) formed by their secretions. The ECM is highly dynamic, comprising secreted molecules making up the basement membrane (BM) and interstitial matrix, comprising proteins that make up the “core matrisome”, including fibronectin, laminins, collagens, elastin, and other glycoproteins [3]. This three-dimensional scaffold, consisting of over 300 different proteins [4], is not passive, as it acts to sequester growth factors and signaling molecules, and promotes the dissemination of information [5,6,7,8,9,10]. Further, its components can act as ligands for cell receptors, providing both biochemical and mechanical signals to cells that modulate cell behavior [11].

The ECM is constantly being remodeled through processes such as synthesis, degradation, reassembly, and chemical modification. This process is complex and tightly regulated to maintain tissue homeostasis, but when dysregulated, it can exacerbate disease progression, including promoting cancer initiation, progression, and dissemination. Notably, the mass of solid tumors consists of up to 60% of ECM deposits, containing fibronectin, elastin, laminin, and collagen [12]. It is evident that differential gene expression of ECM proteins compared to normal tissue exists across cancer types, including hepatocellular carcinoma [13], head and neck squamous cell carcinoma [14], high-risk cutaneous squamous cell carcinoma [15], retinoblastoma cerebrospinal fluid [16], colorectal cancer [17], and brain metastatic breast cancer [18]. For example, mammographically, this is apparent, as dense breast tissue associated with increased collagen-matrix density is a great risk factor for cancer development [19]. Also, in papillary thyroid cancer, firm and hard nodules upon palpation are accompanied by calcification and fibrosis, and more collagen in the stroma increases the strain ratio on ultrasonography [20].

In the appropriate context, the ECM would be sufficient to restrain cancer progression. However, the tumor cells and host stroma co-evolve over the course of cancer progression [21,22]. It is suggested that the differentiation from stromal cells to tumor-associated stromal cells occurs and facilitates cancer progression [23,24,25]. These stromal cells populating the TME include, but are not limited to, blood and lymphatic endothelial cells, mesenchymal stem cells, and cancer-associated fibroblasts (CAFs). It has been proposed that the most aggressive cancer-associated stromal cell phenotypes are those involved in matrix remodeling, largely induced by CAFs [21,26,27]. CAFs are a heterogeneous cell population within the TME, distinguished from normal fibroblasts by increases in fibroblast-activating protein (FAP), α-smooth muscle actin, vimentin, and collagen type I α2 (COL1A2). Their activation enables the formation of the abnormal ECM deposition and stiffness observed in cancer and fibrosis [28]. CAF synthesis of ECM proteins, including collagens, results in characteristic desmoplasia [29]. Desmoplasia is a significant stromal alteration in cancer, as it is the dense connective tissue growth resulting from tumor progression, such as fibrotic tissue [30]. ECM stiffness, due to an increase in collagen deposition and crosslinking, contributes to tumor progression, disrupting tissue morphogenesis [31].

The stromal cells in the TME, which are responsible for this phenomenon, are activated by signals from tumor cells and other stromal cells include cytokines and growth factors, such as transforming growth factor (TGF)-β1, epidermal growth factor (EGF), platelet-derived growth factor (PDGF), fibroblast growth factor (FGF)-2, and C-X-C motif chemokine ligand (CXCL)-12 [32,33,34]. Cell-derived factors, including microRNAs (miRNAs) and exosomes, have also been implicated in the formation of cancer-associated stromal cell subtypes through their recruitment and modification. miRNAs are small, single-stranded noncoding RNAs that epigenetically regulate various physiological processes implicated in cancer. Their expression has been demonstrated to be altered in cancer-associated cells, impacting their activation and signaling cascades, including MAPK (Mitogen-activated protein kinase), also altering the ECM [35,36,37,38]. For example, miR-26b downregulation in breast cancer CAFs results in upregulation of collagen type XII α1 (COL12A1), enhancing migration and invasion of the cancer cells [39]. Further, downregulation of miR-29a/b in pancreatic cancer results in upregulation of collagen type III α1 (COL3A1) and TGF-β1 [40]. Exosomes are membrane-enclosed microvesicles that contain molecules of their origin cell, including RNA, DNA, and proteins, and those derived from cancer cells can both induce CAF formation and regulate the secretion of proteins involved in ECM regulation and tumorigenesis [41,42]. Cancer cell-derived exosomes contain TGF-β, which activate CAFs through induction of SMAD (Sma- and Mad-related protein) signaling [43]. Synergistically, this results in a TME that is inflamed, immunosuppressive, and tumor promoting [44,45]. While different across cancer types, this is generally characterized by leaky vessel vascularization, increased blood flow and interstitial pressure, recruitment of various immune cells, and increased ECM and collagen deposition [46,47].

Collagens and their regulatory enzymes have been shown to be differentially expressed in various types of cancer [48]. Collagen fiber organization 10–20 cm away from a malignant tumor has been shown to be completely altered [49], and changes have been shown to be stage-dependent [50,51]. There are five parameters of collagen, including alignment, density, width, length, and straightness [52]. Interestingly, collagens can both promote or prevent tumor progression along the metastatic cascade, influenced largely by cancer type and stage [53]. This narrative review describes the cellular and molecular mechanisms whereby collagens serve as barriers to tumor spread or facilitate metastasis at the level of the primary tumor site and metastatic niche.

## 2. Collagen Biosynthesis and Signaling

Collagen is the most abundant protein in the human body, accounting for about one-third of the body’s total protein and three-quarters of the dry weight of human skin [54]. Collagens are the primary building blocks of skin, muscles, bones, tendons, ligaments, and other connective tissues. They function by aiding fibroblasts in dermis formation, replacing dead skin cells, protecting organs, aiding in blood clotting, and contributing to skin strength, structure, and elasticity [55,56].

Collagens are glycoproteins consisting of triple helix chains characterized by glycine-X-Y repeats without interruptions, of which X and Y are usually proline or hydroxyproline [57]. The assembly of the triple helical structure begins at the C terminus of nascent chains and forms procollagen [53]. To ensure correct alignment, this is accompanied by chaperone proteins to ensure correct alignment, including heat shock protein (HSP)-47, prolyl-hydroxylase (PH), and protein disulfide isomerase [58]. Following translation, hydroxylation and glycosylation occur in the endoplasmic reticulum (ER), which is regulated by both vitamin C and pyruvate metabolism [53]. Hydrolysis of procollagen to form collagen is performed by N- and C-terminal proteinases surrounding the ER. In the presence of calcium, these enzymes catalyze the removal of collagen propeptides, with HSP-47 and protein disulfide isomerase. Endopeptidases and metalloproteases can also excise procollagen at both the N- and C-termini [58].

Collagens are localized to the plasma membrane or released into the ECM based on the fibril supramolecular assembly formed, which begins when trafficking from the Golgi to the membrane. This assembly is stabilized by intra- and inter-molecular crosslinking, mostly covalently, through lysyl oxidase (LOX), LOX-like (LOXL) enzymes, glycosylation, and transglutaminase crosslinks, depending on collagen type. There are 28 types of collagens (I-XXVIII), some with several isoforms, identified in human tissues. These triple-helical domain-containing proteins are classified by their supramolecular assembly and non-collagenous protein domains [58]. These include fibrillar, non-fibrillar, fibril-associated collagens with interrupted helices (FACITs), network-forming, membrane-anchored, multiplexins, beaded-filament forming, and “others”. Fibrillar collagens consist of one major triple-helical domain and assemble into long, strong fibrils that serve as structural support in tissue [58]. Collagen types I, II, III, V, XI, XXIV, and XXVII are fibrillar, with I and III being the most abundant in the interstitial matrix [58]. Collagen types IX, XII, XIV, XVI, XIX, XX, XXI, and XXII are FACITs, influencing fibrillar collagen shape and thickness [58]. Collagen types IV, VIII, and X are network-forming, with collagen type IV being a major BM constituent [58]. Membrane-anchored collagens are XIII, XVII, XXIII, and XXV, and are the major source of matricryptins, cleaved collagen fragments with biological activity [58]. Collagen types XV and XVIII are multiplexins (endostatin-producing), which also serve as matricryptins, and collagen type VI is a beaded-filament-forming collagen [58]. All of the non-fibrillar collagens have several triple-helical domains and vary in their non-collagenous domains, hence providing these various functions. “Others” that do not fit well into any category include collage types XXVI and XXVIII.

The collagens that constitute the BM beneath epithelial and endothelial cells include IV, VI, VII, XV, VIII, and XVIII [59], with roles influencing cell behavior. Collagens I, II, and III are present in the interstitial matrix, between cells in tissues, and serve as a main structural framework, enabling cell movement and attachment [60]. Collagens type I, III, and IV also serve as matricryptins, with their levels being age- and sex-dependent and implicated in chronic inflammation and fibrosis [60]. Collagen production decreases with age, and existing collagen begins to break down at a much faster rate [55]. Notably, collagen production significantly decreases in women after menopause, but everyone experiences a decline after 60 years of age [60,61]. In addition to hormones, this change is due to external factors over time, such as ultraviolet radiation, pollution, and a poor diet [62,63], resulting in both a decrease in fibroblast activity and an increase in collagen breakdown [64]. Hormone replacement therapy [65,66] and collagen supplementation have been shown to be beneficial [67,68].

Collagen biosynthesis is regulated at the transcriptional and translational level, and this change is accomplished by noncoding RNAs, targeting their gene expression [69], or the enzymes involved in their processing, including hydroxylation [70] and crosslinking [71]. Further, gene mutations [72], transcription factors [73], and signaling pathways, like TGF-β [74], can affect collagen expression. While the main producers of collagens are fibroblasts and cancer cells, tumor-associated macrophages (TAMs), particularly M2, have demonstrated the ability to produce collagen [75], including collagen type I in bladder cancer [76]. Cell-autonomous ECM production has also been demonstrated in metastatic prostate cancer cells, promoted by CAF-secreted lactate [77]. Interestingly, cancer cells may produce abnormal, unique collagen trimers, termed “oncogenic collagen” by Chen et al. [78]. In this instance, normal collagen type I (α1/α2/α1) is suppressed, and oncogenic collagen trimer α1/α1/α1 is produced by pancreatic cancer cells [78]. Excitingly, oncogenic collagen variants are a novel concept and still warrant further exploration.

Collagens mainly interact with cancer cells through cognate receptors on the cells. Known collagen receptors include integrins (ITGs), discoidin domain receptor tyrosine kinase 1 (DDR) 1, DDR2, glycoprotein VI (GPVI), osteoclast-associated receptor (OSCAR), leukocyte-associated immunoglobulin-like receptor 1 (LAIR-1), LAIR-2, and pro-urokinase-type plasminogen activator receptor-associated protein (UPARAP)/endocytic receptor (ENDO)180 [57]. Downstream of the collagen binding integrins, activation of focal adhesion kinase (FAK) and Src family kinases (SFKs) occurs, which regulates cell behavior through including PI3K/Akt (phosphoinositide 3-kinase/protein kinase B), MAPK (p38, JNK (c-Jun N-terminal kinase), and ERK (extracellular signal-regulated kinase)), and Rho family GTPases (Rac1 and RhoA). DDR1 and DDR2 binding activate SFKs and pathways, including PI3K/Akt and MAPK (p38, JNK, and ERK). GPVI signaling by collagen binding is involved in platelet activation, with downstream activation of PI3K/Akt signaling. Collagen binding to immune receptor OSCAR on osteoclasts triggers signaling pathways, including Rho GTPases. LAIR-1 is an inhibitory receptor of immune cells, with LAIR-2 being its soluble decoy molecule that binds to collagen with higher affinity and activates SFKs. Lastly, UPARAP/ENDO180 also activates RhoGTPases.

**Figure 1:**
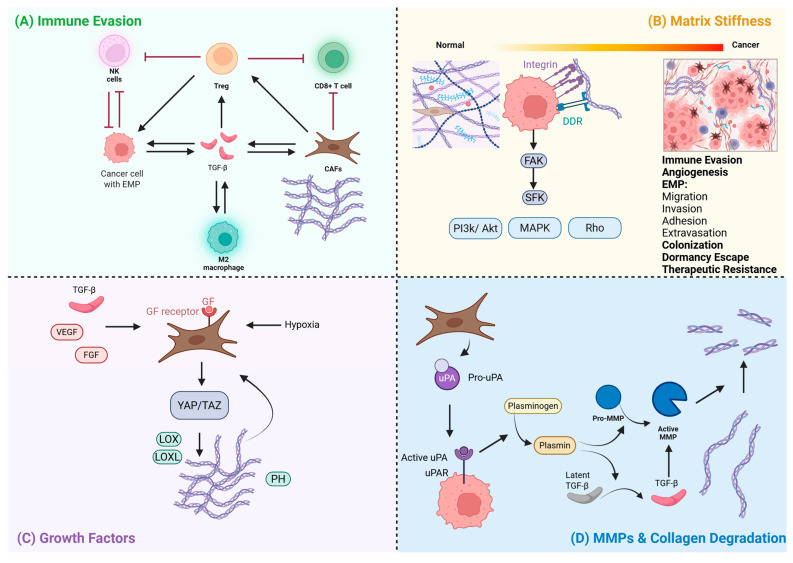
ECM composition and remodeling in the TME. (**A**) The interplay of cancer-associated fibroblasts and collagen deposition and crosslinking in the TME is able to modulate immune system activity through inhibition of cytotoxic T cells and NK cells, promoting T regulatory cells and macrophage activation. (**B**) Cancer-associated fibroblast activation by growth factors and hypoxia results in YAP/TAZ signaling that increases collagen deposition and crosslinking by LOX, LOX-L, and PH enzymes. (**C**) Matrix stiffness and the consequential signaling of crosslinked collagen and its receptors result in downstream FAK/SRC signaling that activates pathways including PI3K/Akt, MAPK, and Rho GTPases, resulting in metastatic phenotypes. (**D**) The activation of the uPA/PAR system and collagenase MMP activation results in collagen degradation in the TME and the production of fragments. CAF = Cancer-Associated Fibroblast, DDR = Discoidin Domain Receptor, EMP = Epithelial–Mesenchymal Plasticity, FAK = Focal Adhesion Kinase, FGF = Fibroblast Growth Factor, LOX(L) = Lysyl Oxidase (Like), MAPK = Mitogen-Activated Protein Kinase, MMP = Matrix Metalloprotease, NK = Natural Killer, PH = Prolyl Hydroxylase, PI3k/Akt = Phosphoinositide 3-kinase and Protein Kinase B, TGF = Transforming Growth Factor, Tregs = Regulatory T cells, uPA/PAR = Urokinase-type Plasminogen Activator/Receptor, VEGF = Vascular Endothelial Growth Factor.

Taken together, functionally, collagens play dual and context-dependent roles in cancer progression, functioning either as physical barriers that limit tumor cell proliferation and invasion, or, when structurally remodeled, as facilitators of tumor dissemination. This duality reflects a dynamic interplay between matrix architecture, mechanical properties, and tumor–stroma interactions, highlighting the ECM as both a suppressor and promoter of metastasis. Not all collagens are carcinogenic, and the same collagen may be carcinogenic in one cancer but not another. Various collagen types differentially regulate metastatic phenotypes of cancer cells due to their structural differences, cognate receptor signaling pathways, and spatiotemporal context-dependent cues, including the remodeling and inflammation that occur as the stage of metastasis progresses (Figure 1).

## 3. Tumor-Suppressive Functions of Collagens

### 3.1. Basement Membrane Integrity

BMs are dynamic and thin ECM structures that support epithelial and endothelial cells while serving as an important structural component of the vasculature. Specialized BMs have roles in selective permeability or in cellular differentiation. Several studies have demonstrated that the disruption of the BM is associated with invasive cancers, as cells need to invade the BM to undergo metastasis [79], through the secretions of cancer and cancer-associated cells. Under these conditions, signatures of coding and noncoding genes that are associated with BM integrity correlated with immune cell infiltration and prognosis [80,81].

As the major BM component, collagen type IV is a particularly dual-roled collagen in the progression of cancer with context-dependent effects, evidenced by both anti-tumorigenic fragments and pro-tumorigenic functions. Angiogenesis, the process of recruiting new blood vessels, is a normal phenomenon that occurs throughout life and is also essential for rapid tumor growth and metastasis. An initially small, avascular tumor undergoes an angiogenic switch, enabling the secretion of angiogenic factors and angiogenesis. These new blood vessels provide the supply of oxygen and nutrients to cancer cells, thereby facilitating tumor growth. This process is controlled by chemical signals that can be secreted by the tumor cells themselves, notably vascular endothelial growth factor A (VEGFA), which binds its cognate receptor [82], resulting in the attraction of endothelial cells that secrete enzymes that migrate or sprout toward existing vessels, proliferate, and morph to form a new vessel [83]. This signal that initiates the growth and survival of new blood vessels is balanced by angiogenesis inhibitors, but is dysregulated in the context of cancer [84]. Notably, in colorectal cancer, collagen type IV α chains are remodeled in the BM, with loss of anti-angiogenic [85] α5 and α6 in the early stage of cancer invasion through hypermethylation of the bidirectional promoter region [86]. Collagen type IV α3 also demonstrates anti-angiogenic functions, inhibiting neovascularization and suppression of tumor growth [87]. Collagen type IV α2 non-collagenous domain inhibits angiogenesis and adhesion of melanoma cells while inducing cell senescence [88].

Laminin is also a large component of the BM, and its dysregulation is also evident during cancer progression, including post-transcriptional modification of laminin-5 in prostate cancer [89], loss of laminin-332 in the early stages of prostate cancer [90], and loss of laminin-1 in breast cancer [91]. Specifically, in pancreatic cancer, immunostaining showed fewer intact BMs with less collagen type IV and laminin staining in invasive carcinomas [92]. This was also seen in colorectal cancer–collagen type IV immunostaining, whereby less staining was associated with shorter survival, and high invasive and metastatic capacity [93]. In summary, BM constituents in normal tissue, especially collagen IV and laminins, serve as barriers that prevent early invasion of cancer.

### 3.2. Anti-Tumor Immunity

A “soft”, or loosely organized, ECM may enhance anti-tumor immune responses by promoting T cell proliferation, activation, and migration. This loosely packed collagen matrix facilitates CD8+ T cell infiltration and supports T cell receptor signaling and cytotoxic functions [94,95,96,97,98,99,100]. When actively softened by inhibition of collagen crosslinking enzyme LOX, CD8+ T cells were infiltrated and motility was increased, and consequently, anti-PD (programmed cell death protein)-1 therapy was enhanced [25]. Additionally, on soft collagen coatings, macrophages were less immunosuppressive, enabling T cell attraction [101]. Collagen type V has been shown to induce an immune response, while increasing endothelial cell apoptosis and preventing angiogenesis in non-small cell lung cancer [102]. In all, less or loose collagen arrangements promote anti-tumor immunity.

### 3.3. Anti-Tumor Induction of Dormancy

Dormancy can occur at the cellular level, whereby transient cell cycle arrest occurs, also called quiescence. Cell viability is still maintained in this reversible non-proliferative state of the cell, but metabolic needs are kept low [103,104]. At the level of the tumor mass, equal rates of proliferation and apoptosis occur, meaning the neoplasm does not progress [103,104]. This mechanism, therefore, slows tumor growth and prevents progression to a more aggressive tumor. Research suggests that this complex process is regulated by intrinsic and autocrine signaling, as well as extracellular signals from supporting cells and the TME [104].

To assess the ECM effects on initial proliferation of lung cancer cells, non-metastatic primary cells were cultured on ECM-derived gels, and cell-cycle arrest was induced due to insufficient ITGβ1-mediated FAK activation, while metastatic cells continued cell cycle progression on the same gels [105]. In breast cancer, tumor-derived collagen type III is required for sustaining dormancy [106]. Collagen type III is also increased in head and neck squamous cell carcinoma patients without lymph node metastasis, and, therefore, it may be manipulated for use as a barrier to metastasis by dormancy induction. Melanoma cells that contact fibrillar collagen type I experience growth arrest with high p27 levels, indicative of dormancy [107]. On non-fibrillar collagen, the same cells continued through the cell cycle and had downregulated p27 expression, indicating no dormancy and confirming context-dependent effects of collagens on the regulation of dormancy.

Dormancy has also been observed with an increased density of immune cells, including metastasis-associated macrophages [108], CD8+ T cells [109], and NK cells [110]. Additionally, stromal cells and other ECM components can provide chemical, physical, and mechanical signals that control dormancy of tumor cells, including, for example, TGF-β2 signaling [111]. Hence, these dormant cancer cells may also be restrained by the immune system.

**Figure 2:**
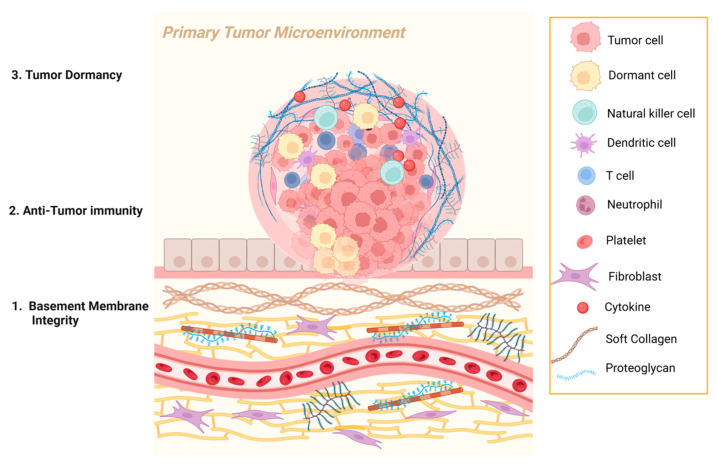
Tumor-suppressive effects of collagens. At the primary tumor site, collagens can play a role in the suppression of tumor progression, including through maintaining basement integrity, providing signals for anti-tumor immunity of cytotoxic T cells and natural killer cells, and establishing tumor dormancy.

Therefore, with the loss of BM integrity by degradation, and transition from a “soft” ECM, characterized by curly and loosely organized compliant collagenous proteins, to a “stiff” ECM, characterized largely by stiff and crosslinked collagen, the tumor matrix promotes dormancy escape and other metastatic phenotypes of the tumor cells (Figure 2).

## 4. Pro-Metastatic Functions of Collagens

**Figure 3:**
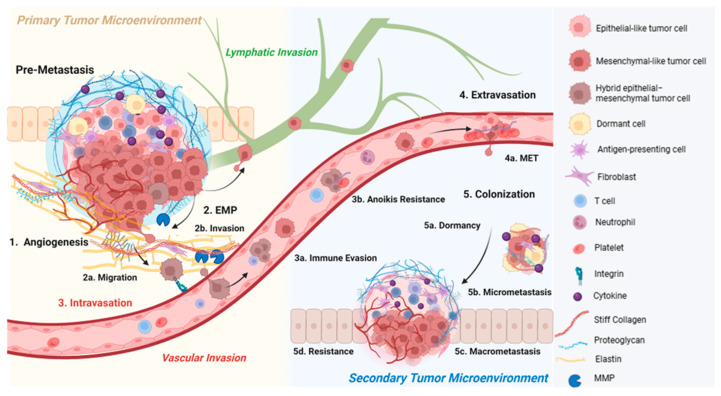
Tumor-promoting effects of collagens in the metastatic cascade. Collagens serve important functions at both the primary tumor site and the metastatic niche of the secondary tumor microenvironment. Collagens have roles in cancer progression through the promotion of angiogenesis; epithelial–mesenchymal transition, and hence, migration, adhesion, and invasion; survival in circulation; extravasation and colonization at distant sites; and dormancy re-awakening. CTCs—circulating tumor cells; EMT—epithelial–mesenchymal transition, MET—mesenchymal–epithelial transition.

Once the cancer cells have circumvented the infrastructure set forth to limit cancer progression, the cells that established a primary tumor can develop metastatic phenotypes. Metastasis begins with the induction of angiogenesis, which provides the vasculature required for nutrients and a route of invasion. Invasion is further promoted by epithelial–mesenchymal plasticity that is characteristic of metastatic cells, enabling adhesion, migration, and local invasion. Vascular invasion can then occur, as single or collective circulating tumor cells (CTCs), into the circulation. Lymphatic spread has fewer phenotypic requirements than vascular spread, as there is no BM that requires protease degradation, since the function of the lymphatics is to constantly sample the environment to combat infection and eliminate waste. In lymphatic spread, migration results in metastasis in the draining lymph nodes. Once in circulation, these cancer cells must survive continued measures that would prevent metastatic spread, including the harsh conditions of the vasculature, anoikis, and the immune system. Imperative to distant metastasis, the cells must extravasate and colonize at a secondary site. Just because tumor cells have entered the circulation, it does not guarantee the formation of a secondary tumor. The intricate nature of these processes and the pro-metastatic role that collagens play at each step is described below (Figure 3).

### 4.1. Tumor-Promoting Mechanoregulation

Largely, collagen deposition and alignment play a role in the mechanical characteristics of the ECM. Analysis of colorectal cancer compared to an aggressive obstructive variant revealed substantially different ECM compositions, but mainly collagen, both newly formed and mature, and a higher abundance of CAFs [112]. In Merkel cell carcinoma, textural features, owing to collagens, are profoundly different at the primary tumor site, and may contribute to the aggressive behavior [113]. The stiffness, or resistance to deformation, is a biophysical characteristic of the ECM attributed to collagen content and alignment that gives it tensile strength and rigidity and regulates cancer cell progression by altering cell phenotypes [19,112,114,115,116,117,118]. It has been demonstrated that in breast cancer, an environment that is characterized by non-fibrillar collagen has cells of a less invasive capacity than that of a fibrillar collagen matrix [119,120]. This coincided with compromised cell polarity and protrusions, lower proliferation, and increased apoptosis [118]. Softer ECMs also demonstrate less cell spreading, focal adhesion (FA) formation, and migration of ovarian cancer cells [121], and less cell spreading of non-cancerous human mesenchymal system cells in periodontal tissue [122].

The ECM is also characterized biophysically by viscoelasticity, displaying viscous (fluid-like) and elastic (solid-like) deformation upon stress, which is essential to tissue development, adaptation, and response to mechanical stimuli, whereby collagen alignment also plays a role. The viscoelasticity of the ECM allows for its preserved architecture due to the solid-like state, and its dynamic reorganization due to its viscous-like state, both of which are important for cell migration, tissue morphogenesis, organ development, and cancer progression [123,124,125]. The tumor ECM viscoelasticity arises from covalent crosslinking and strain-stiffening responses of collagen scaffolds, impacting cell cytoskeleton and FAs [123]. For example, collagen architecture and alterations enhance ECM viscoelasticity in hepatocellular carcinoma, promoting its progression in cirrhotic conditions [124]. Even in a non-cancerous context, patient-derived fibroblasts with mutations in collagen type III had altered ECM viscoelasticity [126].

Another biophysical characteristic of the ECM modulated in the context of cancer is interstitial fluid pressure and flow. The increased collagen deposition by contractile CAFs in the tumor stroma results in the dysfunction of blood vessels, which affects interstitial fluid pressure [127]. This dysfunction has been shown to be a poor prognostic factor in some solid tumors [128,129,130], affecting cancer therapy [127], and promoting the detachment of clusters of strongly invasive cells [131].

Biochemically, the ECM functions in dynamic reciprocity, whereby the cell–ECM interactions influence both the properties of the ECM and provide mechanical cues to the cells, including in the context of cancer [19,117,132]. The more dense and rigid ECM has functional roles in cell migration, invasion, and proliferation, while also shielding and nourishing primary tumors [118,123,133]. Favorable matrix stiffness and fluid pressure are structural outcomes of the collagen composition, structure, and remodeling that are attributed to the crosstalk between the ECM and the surrounding cells.

Altered collagen deposition and remodeling in the TME that affects mechanical signaling in cancer cells are largely due to CAFs and collagen remodeling enzymes, and the positive feedback loop that ensues. The activation of Yes-associated protein (YAP) transcriptional co-activator with PDZ-binding motif (TAZ) is essential for YAP function, which is required for CAFs to promote matrix stiffening [134]. Their elevation can be promoted by the stiff matrix and is associated with increased metastasis [135]. YAP has also been shown to be modulated by mechanosensitive kinase ROCK1 (Rho-associated protein kinase 1) and ECM stiffness in endometrial cancer [136].

LOX-mediated collagen crosslinking is responsible for the characteristically increased fibrosis in cancer and tumor progression [117]. LOX expression is associated with lymph node metastasis and significantly lower survival in patients with esophageal squamous cell carcinoma [137]. PHs are responsible for collagen stability and have also been correlated with cancer growth and metastasis in ovarian cancer [138,139]. Additionally, collagen-rich conditions facilitate hypoxia, which further intensifies cancer progression, manipulating cancer cell activity. In high-grade serous ovarian cancer, hypoxic signaling increased LOX expression and enabled a rich fibrillar collagen niche [140]. If transcription factors of hypoxia-inducible factors (HIFs) are silenced, enzymes involved in collagen type I degradation, synthesis, and deposition are altered, including PH and LOX downregulation, coinciding with an increased collagen type I volume [141]. This change was also evident in the context of normal bone formation, whereby inappropriate HIF-1α signaling resulted in collagen over-modification, via hydroxylation, and hence skeletal dysplasia [142]. Mechanistically, HIF-1α activation under hypoxic conditions alleviated Activating Transcription Factor-3 methylation at the promoter and stimulated PH [143].

### 4.2. Primary Tumor Immune Evasion

The ability to evade the immune system is crucial for cancer cell survival. Tumor cells themselves upregulate the expression of molecules that will block T cell activation, including PD-L1 (programmed death-ligand 1) [144] and CD45 [145]. Correlation of PD-L1 expression, CD8+ T cell infiltration, and desmoplasia characterized by dense collagenous stroma have been previously reported [146]. The ECM can serve as both a structural blockage of immune cells [147] and a modulator of immune cells and tumor-associated stromal cells’ phenotype to promote an immunosuppressive environment [148]. Specifically, collagen remodeling that results in reduced pore size is a barrier to prevent immune cell infiltration, but also reprograms the T cells [100]. For example, a stiffer matrix induced CD4+ T cell deactivation [100]. HSP-47-mediated collagen deposition also inhibited CD8+ T cell activation [149]. TAMs have demonstrated TGF-β-directed collagen biosynthesis that caused a stiff and fibrotic TME that exhausted the CD8+ T cells and physically excluded them [150]. Co-inhibition of TGF-β and PD-L1 resulted in decreased collagen deposition, enhanced CD8+ T cell penetration, and increased tumor-infiltration lymphocytes [151]. Other mechanisms of reduced collagen deposition and crosslinking have reduced T-cell exhaustion as well [152], including LOXL2 suppression [153] and YAP inhibition [154].

Viscoelasticity of the ECM also functionally alters T cells, to a higher degree than stiffness in one study, via the Activator-Protein-1 signaling pathway, and imprints long-term on the T cell phenotype [125]. Interestingly, tumor-infiltrating T regulatory cells have demonstrated YAP signaling in response to a stiff matrix, facilitating their immunosuppressive function [155]. Collagen binding to its cognate inhibitor receptors, LAIR-1 and DDR1, also plays a role in immunosuppression. DDR1 expression in triple-negative breast cancer negatively correlated with the abundance of anti-tumor T cells, and its promotion of collagen fiber alignment instigated immune exclusion [156,157,158,159]. Mechanistically, it has been suggested that this is through inhibition of IL-18 synthesis and upregulation of PD-L1 expression by JNK signaling, and hence lower infiltration of CD4+ and CD8+ T cells [160]. Collagen-activated DDR1 resulted in the promotion of the differentiation and immune infiltration of Tregs (regulatory T cells) in breast cancer [161]. This also resulted in the formation of neutrophil extracellular traps (NETs) upon neutrophil degranulation with tumor activation, which promoted Treg infiltration [161]. DDR1-induced NETs are also evident in pancreatic ductal adenocarcinoma, whereby CXCL5 production recruited tumor-associated neutrophils (TANs), through DDR1/NFκB (nuclear factor kappa-light-chain-enhancer of activated B cells) signaling, and consequently promoted metastasis [162], most notably in collagen-dense tumors [163]. Collagen inhibitory receptor LAIR-1 is also implicated in immunosuppression, promoting CD8+ T cell exhaustion through T cell regulator Src homology region 2 domain-containing phosphatase 1 [153]. Collagen-activated LAIR-1, specifically mediated by overexpression of collagen type XVII, has also resulted in diminished NK cell cytotoxic activity [164]. With that, blocking of LAIR-1 binding to collagen has demonstrated augmented cytotoxic T cell infiltration and anti-tumor responses [165,166]. Therefore, collagens play pivotal roles in immune cell infiltration and immunosuppression through collagen receptor signaling pathways that alter tumor-associated stromal cells, such as macrophages, fibroblasts, and neutrophils, and immune cells.

### 4.3. Angiogenesis

Collagens also play a pro-angiogenic role, as endothelial cells must adhere and migrate through the ECM by integrins, and as collagen crosslinking creates an ideal angiogenic scaffold [134]. Binding of ITGαV/ITGAβ3 and ITGαV/ITGAβV to the non-collagenous domain of collagen type IV α3 [167] has been shown to enable angiogenesis. Nitric oxide also mediates collagen type IV synthesis of endothelial cells, contributing to angiogenesis of lung endothelial cells via integrin-FAK signaling [168]. It has been reported that a stable extracellular network of collagen type IV, increasing in secretion and deposition, is required for angiogenesis [169]. Additionally, collagen type I has been shown to stimulate lung tumor cells to secrete VEGFA, and its inhibition suppressed angiogenesis in vivo [170]. It has been demonstrated that in collagen type I, binding to endothelial cells ITGα2/ITGβ1 is required for angiogenesis [171]. Additionally, the crosslinking of collagen plays a role in angiogenesis, as upon treatment of human glioblastoma cell lines with a LOX inhibitor, VEGFA expression and tumor angiogenesis were inhibited [172]. Therefore, collagens play a significant role in angiogenesis.

### 4.4. Epithelial–Mesenchymal Plasticity

As angiogenesis provides nutrients and oxygen for the growing tumor, the cells now have a higher potential to intravasate into circulation. Cancer cells tightly regulate adhesion molecules, like endothelial cells during angiogenesis. Loss of adhesion is triggered upon induction of epithelial–mesenchymal transition (EMT) in cancer cells at the invasive front, which is stimulated by the TME. Characteristic changes that constitute the acquisition of “fibroblast-like” or mesenchymal properties include the loss and gain of the expression of several different related factors. EMT consists of the loss of cytokeratin and E-cadherin expression along with the gain of N-cadherin, vimentin, integrins, and PDGF receptor expression. These cells lose their epithelial cell polarity and gain a fibroblast-like shape while also secreting proteases and fibronectin [173,174]. The loss of tight and adherens junctions accounts for the reduction in cell–cell adhesion; meanwhile, the expression of mesenchymal genes results in the acquisition of cell motility, invasion, cell–matrix interactions, and matrix production. EMT has been reframed, as this is rarely binary, with cancer cells demonstrating the intrinsic ability to interconvert as a hybrid epithelial and mesenchymal state, known as epithelial–mesenchymal plasticity (EMP). EMT is therefore considered a spectrum, with cells more characteristic of a partial EMT (pEMT). This intermediate population of cells is more effective at undergoing metastasis, facilitated by collective migration and survival signals that could otherwise be lost upon detachment from the ECM and released into circulation. This hybrid phenotype is believed to have the ability to resist anti-cancer therapeutics [175]. It has been demonstrated that pEMT is controlled by EMT transcription factors that are regulated by exosomes, soluble factors, and the ECM remodeling and subsequent alterations in its composition and mechanics [176].

EMT is triggered by stromal signals, including FGF, hepatocyte growth factor, TGF-β, wingless-related integration site (Wnt), and tumor necrosis factor (TNF)-α. This is also accompanied by the subsequent upregulation of EMT transcription factors, including FOXC2, SNAIL (SNAI1), TWIST, and SLUG (SNAI2) [173,174]. For example, in response to TGF-β signaling, SMAD3/4 complexes activate SNAIL, ZEB1, and TWIST to affect the expression of epithelial and mesenchymal genes [177]. In a non-small cell lung cancer model, researchers induced the expression of TGF-β, and the transcription factors it and other EMT-inducing ligands converge on SNAI1 and ZEB1 [178]. The induction of TGF-β, SNAIL, and ZEB1 all resulted in characteristic EMT; however, the TGF-β-induced EMT model caused a more complex EMT with more pronounced changes in ECM remodeling [178]. In breast cancer, the mechanosensitive Epha2/Lyn protein complex is phosphorylated upon high ECM stiffness and results in TWIST release to the nucleus [179]. In colorectal cancer, high ECM stiffness also regulated EMT-related signaling pathways through HSP-47, including E-cadherin, N-cadherin, and β-catenin [115]. In 3D co-culture models of lung adenocarcinoma cells and TAMs, increased tumor stiffness enhanced tumor cell invasion, increasing N-cadherin and TWIST1 expression [155]. When investigating the effect of collagen prolyl hydroxylation, its inhibition blocked metastatic dissemination of breast cancer cells by preventing EMT [180]. In agreement with this result, inhibition of DDR2 signaling decreased collagen production, SNAI1 protein expression, and invasion [181,182,183] that normally would be activated through downstream PI3K/Akt signaling upon collagen type I stimulation [184]. This was also seen in papillary thyroid cancer cells as DDR2 overexpression promoted EMT through SNAI1 protein expression [185]. EMT transcription factor ZEB1 has also been shown to promote EMT through regulation of LOX and LOXL2 to stabilize collagen crosslinks and promote mesenchymal signaling [186]. Collagen types I, X, and VIII also demonstrated the ability to facilitate EMT in tumor cells, promoting metastasis across various cancers [187,188,189,190].

It is evident that tumor cells retaining both epithelial and mesenchymal characteristics adapt to stress and respond to environmental cues in a superior manner to a fully epithelial or fully mesenchymal phenotype. The ability of the cancer cell to modulate this along the metastatic cascade is imperative to the successful formation of a distant metastasis, beginning with the acquisition of mesenchymal phenotypes enabling invasion and migration.

### 4.5. Migration

Dysregulation of tight and adherens junctions occurs during EMT induction, and it is imperative to the migratory capacity of the cells. In addition to the loss of these molecules during EMT, the molecules that mediate ECM interactions, including integrins, laminin receptors, and CD44, become upregulated. CD44 mediates tumor attachment to the ECM component hyaluronic acid and is strongly associated with cell motility and metastasis [191]. While laminins and integrins can bind to each other, laminins can also bind to ECM components, including collagens, syndecans, and heparins. Integrins can also bind ECM components such as collagens and fibronectins, resulting in PI3K/Akt, MAPK, and Rho signaling to modulate cell adhesion [57].

Integrins are heterodimeric and mediate cell adhesion by acting as the “velcro” of the cell. Integrins control actin fibers that allow for ruffling of the membrane and movement in specific directions, micrometers at a time. In addition to integrin activation, receptor tyrosine kinases (RTKs), G-protein-coupled receptors, cytokine receptors, and Wnt and Notch signaling converge to activate Rho GTPases through guanine nucleotide exchange factors [192]. Rho GTPases act as binary switches, similar to Ras, regulating cell motility through the constant cycling of phosphorylation events, resulting in active (GTP-bound) and inactive (GDP-bound) conformations [192]. These comprise three subfamilies: Rho, Rac, and Cdc42, all involved in protrusive actin filament networks and associated integrin complexes, including stress fibers, lamellipodia, filopodia, and FA [193,194,195,196,197].

Integrins, therefore, play important roles in cell migration. For example, pancreatic cancer-produced collagen type IV colocalizes with integrin receptors of pancreatic cancer cells and simulates an autocrine signal that promotes migration and proliferation while inhibiting apoptosis [198]. In triple-negative breast cancer, ITGα3/ITGAβ1 is an important regulator of invasion and metastasis [199]. Further, when blocking collagen receptor ITGβ1, tumor cell migration significantly decreased [200].

Integrins also constitute specialized structures called hemidesmosomes that facilitate adhesion of epithelial cells to the BM by anchorage of keratin cytoskeletal intermediate filaments [201]. In addition to integrins, these consist of collagen type XVII, plectin, and other proteins, with specifically ITGα6/ITGβ4 acting as a signaling molecule [202]. In prostate cancer, these hemidesmosomes are lost and associated with actin-rich adhesions, leading to EGFR (Epidermal Growth Factor Receptor)/PI3K/Akt- and FAK/SFK-pathway activation [203].

In addition to generating the force for migration, actin filaments function to sense and translate mechanical signaling of the ECM into signaling events. Increased collagen matrix density promoted both ovarian and breast cancer malignancy through FAK-Rho-ERK signaling [10,19]. These protrusions are also stabilized by adhering to the ECM, as FAs form at sites of ECM contact with the cell membrane, allowing for a physical connection to the actin cytoskeleton, and the conversion of the force generated by actin polymerization into forward migration. Cadherin-mediated adherens junctions also play a role, controlling the position of FAs and the recruitment of proteins involved in cell polarity and migration, thereby regulating lamellipodia activity, cell polarization, and migration direction [204].

Collagen fiber density has been demonstrated to promote tumor adhesion in metastatic ovarian cancer, as opposed to collagen-specific interactions alone [205]. Collagen content, alignment, and distribution affect cancer progression and correlate with clinical outcome [53]. As cancer progresses, collagen will exhibit different signatures, termed tumor-associated collagen signatures (TACSs). There are five parameters of collagen, including alignment, density, width, length, and straightness [52]. There are inherent differences in baseline architecture depending on the tissue of origin, including the type and amount of collagen present, which will have an impact on the TACS. As the cancer progresses and modulates the TME, remodeling enzymes and CAFs will affect collagen phenotypes. TACS-1 indicates dense collagen near the tumor, TACS-2 indicates collagen fibers parallel to the tumor edge, and TACS-3 indicates radially aligned collagen fibers [52]; this leads to guiding the migration of cancer cells to initiate metastasis [206,207]. This anisotropic topography enhances the persistence and velocity of migratory cancer cells up the stiffness gradient, termed durotaxis, hence promoting tumor progression [30,208]. Contact guidance fidelity via local collagen alignment is apparent, with diversity that may be attributed to ROCK signaling [209,210]. TACS-3 correlates with cell invasion and poor survival in breast cancer, as it was shown to be driven by ERK signaling and promoted cell migration [211]. Therefore, increased density and depth of collagen correlate with increased invasion and proliferation of cancer cells.

Amoeboid cell migration may also occur, which relies on shape changes rather than adhesion mechanisms and is instead propelled by actomyosin contractility of pseudopodia or blebs. While mesenchymal migration relies on strong matrix adhesion and an elongated spindle-like cell shape, amoeboid migration demonstrates minimal cell–matrix adhesions and a rounded cell shape. The adaptability of cancer cells to switch between protease-dependent mesenchymal and protease-independent amoeboid invasion is coordinately regulated by pigment epithelium-derived factor and downstream modulation of RhoA and Rac1 [212]. Amoeboid migration also plays a role in cancer metastasis and is shown to be influenced by collagen in the TME. Specifically, collagen type III has demonstrated the ability to promote pseudopodia formation and migration [213]. In conditions whereby cell–matrix adhesions are impeded by modulating collagen concentration and mechanical stiffness, amoeboid phenotypes are induced [214,215]. With the inhibition of LOX and, consequently, altered collagen crosslinking, Endo180-dependent amoeboid-like migration is reduced [216]. In another study, compared to pure collagen gels, glioblastoma cells with increasing hyaluronic acid content and decreasing collagen content displayed a transition from mesenchymal to amoeboid migration [217].

Collective or clustered, migration constitutes a mixed cancer population, characterized by pEMT, and produces a more successful metastatic phenotype [218,219,220,221]. In spheroids, mesenchymal marker vimentin was necessary for collective migration in a 3D collagen matrix [222]. Additionally, supporting the success of a mixed phenotype, the retention of epithelial marker E-cadherin promotes collective cell movement in gastric, ovarian, and mammary cancer cells [223,224,225]. Essential to collective migration, maintaining cell–cell adhesion has been shown to require collagen transmembrane receptor DDR1 through the Par protein complex, which regulates cell polarity [195,226]. Collagen receptor ITGβ1 has been shown to play a role [227]. These collectively migrating cells are not independent cells grouped together, moving at the same speed and direction; instead, they are in communication both mechanically and chemically [228]. These cells can influence each other’s behavior, resulting in more efficient migration. Rho GTPases have been implicated in collective cell migration, as protrusions are present on the leading cells of collectively migrating groups, but not on the cells behind [195]. Specifically, leader cells have higher mechanical interactions and have been shown to respond to aligned collagen to direct collective migration [229].

While enhanced migration through mediation of cell–cell and cell–matrix interactions is important in the metastatic cascade, for dissemination into circulation, tumor invasion, and downstream cell survival must occur.

### 4.6. Invasion

Tumor invasion is another consequence of EMP, whereby the mesenchymal phenotype promotes the lysis of matrix proteins by particular proteases, enabling the translocation of cells across ECM barriers and further promoting cell migration [1,2]. The invasive capacity of cancer cells has been demonstrated to be owed to dysregulation of ECM gene expression [230,231], collagen stiffening [24], crosslinking [143], and glycation [232] across a variety of cancer types. Mechanistically, this has been demonstrated in breast cancer to be owed to the signaling accompanied with collagen crosslinking, stiffening the ECM, promoting the formation of FAs and downstream PI3K activity and invasion by enhancing integrin signaling [117]. In a stiff matrix, the positive feedback loop of YAP activation in CAFs promotes a stiffer matrix, and hence further regulates cancer cell invasion [134,233]. The ITGβ1-tensin-1-YAP mechanotransductive pathway has also been implicated in the invasion of hepatocellular carcinoma cells, activated by the matrix viscoelasticity [124].

The acquisition of invasive phenotypes of cancer cells, established by the collagen crosslinking propagated by CAF activation, results in cancer cells with invasive structures that secrete ECM-degrading proteases. Proteases include various matrix metalloproteases (MMPs), adamlysins, consisting of a disintegrin and metalloproteases (ADAMs), and the pro-urokinase-type plasminogen activator/pro-urokinase-type plasminogen activator receptor (uPA/uPAR) proteolytic system. The cellular changes that promote the invasive and metastatic abilities of cancer cells result in the degradation of the BM, modulation of adhesion, increased cell migration, and resistance to anoikis. The protease activity of matrix-degrading enzymes is critical to invasion and metastasis, as the degradation of the protein barriers in the ECM is necessary for relevant migration and angiogenesis to occur. MMPs serve as liberators of angiogenic factors, regulators of cell adhesion and migration, and processors of growth factors and cytokines [234]. For example, MMPs can cleave E-cadherin between tumor cells and integrins between tumor cells and the ECM. MMPs can also process growth factors like VEGF and FGF, making them more accessible [234]. MMPs can also mediate tumor evasion from T cells through MICA/B lysis [234].

MMPs comprise 16 members, subdivided into four groups based on their structural characteristics and substrate specificities [235]. The soluble and secreted groups include collagenases, gelatinases, and stromelysins, whereas the membrane-type MMPs (MT-MMPs) are anchored to the plasma membrane. In addition to cancer cells, MMPs are produced by tumor-associated cells, including macrophages, neutrophils, endothelial cells, and fibroblasts. These metalloproteases are calcium-dependent and contain a zinc ion in the active center, which is required for their catalytic activities. MMPs are synthesized as inactive proenzymes, which are activated by cleavage of a propeptide [236]. Stromal cells secrete inactive uPA, which binds to its receptor on the cancer cell, uPAR, resulting in its activation and subsequent cleavage of plasminogen to plasmin. Plasmin activates TGF-β1 from its latent form and additionally cleaves pro-MMP to MMP, thereby activating its ability to degrade the ECM. MMP expressions are controlled in two ways: by an increase at the transcriptional level, as well as their ability to activate each other. MMP activity is negatively regulated by specific inhibitors called tissue inhibitors of metalloproteases (TIMPs). TIMPS form non-covalent complexes that block the MMP active sites [237].

Sharing the metalloprotease domain with MMPs, ADAMS, and ADAMS, with a thrombospondin motif (ADAMTS), are closely related MMP families of molecules regulating various biological events, mainly extracellular domain shedding mediation [238]. They can induce proteolytic processing to release membrane-associated proteins and activate molecules involved in growth factor signaling, cell adhesion, and cell migration, among other processes [239]. ADAMS expression is dysregulated in various cancers and promotes proliferation and angiogenesis through the modulation of intercellular adhesion and ECM degradation. Some examples include the release of TGF-β, which subsequently activates EGFR, and in turn, MAPK signaling, leading to the overexpression of MMP-2 and MMP-9 [239]. MMP-2 and MMP-9 are believed to be of the most importance in tumorigenesis [240,241], as they cleave components of the ECM, including collagen type IV, the principal constituent of the BM [238]. Consequently, they have a high correlation with metastasis across various cancer types [242]. Other MMPs have also demonstrated major roles in various cancers, including MMP-3, which has been shown to cause EMT through the increase in ROS [243]. MMP-3 levels in plasma may be a marker of residual metastasis following surgery in invasive colorectal cancer [244]. Additionally, MMP-11 has been associated with a higher incidence of lymph node metastasis (LNM) and lower survival rates of oral squamous cell carcinoma, mechanistically through FAK/SFK signaling [245]. MMP-14 expression enhances gelatin and degradation and invasion in breast cancer cells [246]. Hence, MMP activity is crucial for the metastatic capacity of tumor cells.

MMPs are classified into different groups, with one being collagenases that degrade collagen. This subfamily constitutes interstitial collagenases MMP-1 (Collagenase-1), MMP-8 (Collagenase-2), and MMP-13 (Collagenase-3), and MMP-2, primarily a gelatinase, that also exhibits collagenase activity [247]. In laryngeal [248,249] and thyroid cancer, MMP-1 expression and activity are upregulated [250,251]. In oral squamous cell carcinoma, MMP-1 expression is higher in invasive tumors, suggesting its role as a prognostic indicator [252]. Inhibition of MMP-1 secretion in keratinocytes prevented their collagen degradation [253], and its dysregulation in cutaneous squamous cell carcinoma and chondrosarcoma prevented cell invasion [254,255]. Inhibition of TGF-β signaling and downstream reduction in MMP-1 and MMP-13 synthesis resulted in inhibition of collagen degradation and invasion of cutaneous squamous cell carcinoma [256]. MMP-13 expression is upregulated in laryngeal [249], oral squamous cell carcinomas [257], and its expression in fibrosarcoma [258] and cutaneous squamous cell carcinoma promoted cell invasion [254,259]. MMP-13 upregulation was also demonstrated in head and neck squamous cell carcinoma and was shown to be promoted by SMAD signaling [260].

High serum levels of MMP-8 are associated with poor prognosis in colorectal cancer [261]. MMP-8 overexpression is also evident in ovarian cancer, regulated by IL-1β, and strongly associated with tumor grade and stage, promoting the invasive potential [262]. MMP-2 has also demonstrated pro-invasive effects in thyroid cancer [263] and cutaneous squamous carcinoma [259] with the promotion of metastatic outgrowths in breast cancer models [264]. In hepatocellular carcinoma patients, MMP-2 and its inhibitor TIMP-2 levels in the serum could predict poor prognosis after treatment [265].

Collagenases MMP-12 and MMP-13 are regulated at the site of invadosome formation, mediated by ROCK-II in colon cancer [266]. Invadosomes are a specialized adhesive structure of the cell membrane that is responsible for collagen cleavage [267,268], which is induced by collagen [269,270]. MMP-14 has also been established as a master MMP in the invadosomes of invading cancer cells [271,272]. Invadosomes are also induced by growth factors, cytokines, and integrin signaling, as well as acidic pH, matrix rigidity, hypoxia, and reactive oxygen species (ROS) [273]. Arising from the ventral surface of the cell membrane, this structure enables the secretion of proteases in conjunction with the cell’s forward movement through the dense ECM. WASP (Neural Wiskott–Aldrich Syndrome Protein)/N-WASP activates Arp2/3, actin filaments are elongated and bundled by fascin, and proteases are transported to the tip by vesicular trafficking requiring ARF6 [195]. Invadosomes also harbor a characteristic ring structure of packed adhesion and scaffolding proteins, including FAK and tyrosine kinase structure 5/SH3 domains [273,274]. ITGαV/ITGβ3 binding has been implicated as being essential for collagen-induced invadopodia extension and haptotaxis in breast cancer [270]. A dense fibrillar collagen network has been shown to potently induce invadosomes [275,276], with DDR1 imperative for its formation and degradation ability [277]. The viscoelasticity of the ECM also plays a role in invadosome protrusions, enabling them to extend mechanically and plastically open up channels and migrate through [132]. Hence, invadosomes play a significant role in cancer invasion and metastasis. These protrusions indicate that the induction of EMT and modulation of actin polymerization signaling are relevant to the localized invasion and intravasation of tumor cells during the metastatic process.

The cleavage of full-length ECM proteins by proteases can generate biologically relevant fragments that can promote angiogenesis, facilitate branching morphogenesis, and release growth factors and other active molecules [6]. Collagens are degraded by MMPs, as described above, but also cathepsins, proline oxidase, or sheddases, which results in the release of ectodomains of the membrane-bound collagens [6]. It has been demonstrated that digested collagens expose neoepitopes, and these domains, fragments, metabolites, and telopeptides can play prognostic and physiological roles [6,278,279,280,281,282,283]. Cleaved collagen type I has opposing effects to intact collagen type I, activating DDR1-NFκB-p62-NRF2 signaling, promoting tumor growth and survival, while intact collagen type I triggers DDR1 degradation in pancreatic ductal adenocarcinoma [284]. The cleaved C5 domain of collagen type VI α3 [285] and the non-collagenous domain of collagen type IV α1, termed arresten [286], play opposing roles in angiogenesis, activating and inhibiting it, respectively. Functionally, cleaved collagen fragments serve roles in immune suppression, mediating T cell suppression through LAIR-1 [287]. In all, functionally, the cleaved collagen fragments play imperative roles in the progression of cancer.

### 4.7. Survival in Circulation

Following invasion and migration, intravasation into the bloodstream and metastasis can potentially occur. Intravasation during metastasis has been shown to be directed by oriented collagen fibers [288]. Once cancer cells have intravasated into the bloodstream, cell survival is essential, as mechanical stress due to the fluid shear force of blood flow and the deformation in organ microvasculature is presumed to eliminate a considerable amount of tumor cells [289,290,291]. Mechanistically, upon fluid shear stress, cells that did not undergo apoptosis activated EMT via JNK signaling, promoting their survival [292]. Besides fluid shear force, this harsh environment is further attributed to the lack of adhesion to the ECM and immune system attack. The tumor cells that survive have gained adaptations that lead to their metastasis, including the evasion of the immune system and resistance to anoikis, mechanisms that are both cell autonomous and through the interaction with other cells, including platelets.

#### 4.7.1. Clustered Circulating Tumor Cells (CTCs)

Only about 0.1% of single CTCs can withstand the harsh environment in circulation, with even fewer able to establish distant metastases [293]. Commonly, circulating tumor cells retain epithelial markers like EpCAM (Epithelial Cell Adhesion Molecule), CD45, and cytokeratins (KRT8, KRT18, KRT19) and are phenotypically identified as those that adhere and invade collagen [294,295]. In breast cancer models, a collagen-dense environment significantly increased CTC and the number and size of metastases [296]. Expression of EpCAM and FAPα, also known as seprase, are selection markers of a particularly invasive and metastatic CTC subpopulation [297]. There is a subpopulation of CTCs characterized by a mesenchymal phenotype lacking EpCAM, following the induction of EMT, and the acquisition of vimentin [298]. In high-risk prostate cancer, this CTC subtype can promote cancer progression through EMT and collagen type I α1 expression [299].

Tumor cells that enter the bloodstream as clusters, undergoing collective migration, allow some cells to have less exposure to mechanical stress, thereby increasing their survival and consequently increasing their metastatic potential in circulation [300]. CTCs can cluster exclusively as tumor cells (homotypic) or with other cell types (heterotypic), and both yield enhanced proliferation and survival in circulation, perpetuating the metastatic potential of the tumor cells [301]. The combination of the hypoxic environment and cell adhesion molecules, including plakoglobin, CD44, and claudins, maintains cluster formation [300,302]. DNA methylation, including hypomethylation of Oct-4 and Sox-2, enables CTC cluster formation, and their methylation results in the dissociation into single cells [303]. Heterotypic clusters include those with neutrophils and/or platelets, termed circulating tumor microemboli (CTM), whereby an increased metastatic seeding is observed [304].

Platelet aggregation and activation markers are increased in ovarian cancer patients compared to healthy controls [305] and have been correlated with decreased survival in esophageal squamous cell carcinoma patients [306]. Ovarian cancer cells of different metastatic potential evidently adhere and activate platelets differently; however, platelets and their secretions promote angiogenesis and survival of ovarian cancer cells [307]. Breast cancer cells also alter platelet phenotypes, resulting in VEGF, thrombospondin-1, and TGF-β1 secretions, promoting angiogenesis [308]. Platelets also induce an invasive mesenchymal phenotype in tumor cells through the release of TGF-β and the activation of SMAD and NFκB [309]. In agreement with this, high platelet counts are potentially adversely prognostic in anaplastic thyroid cancer, as they promote cancer invasion and migration through NFκB signaling, resulting in MMP-1 production [310]. Collagens have been shown to induce platelet activation and aggregation in a proteasome and NFκB-dependent manner [311]. Collagens activate platelets through their receptor GPVI, which has been shown to facilitate metastasis [312,313,314]. When platelets are in circulation, ADAM10 cleaves GPVI [315]; however, when spread on collagen, GPVI clusters and sustains Syk signaling for adhesion [316,317,318,319] and enables platelet aggregation [320]. Platelet-derived extracellular vesicles generated with collagen stimulation and subsequent GPVI activation increased melanoma spheroid growth and invasion, altering signaling in pathways involved in ECM organization, protein targeting/processing in the ER, miRNAs in cancer, MAPK signaling, and PI3K/Akt signaling [321]. Inhibition of collagen-induced platelet aggregation inhibits cancer cell growth and proliferation [322]. Hence, physical protection through the formation of microaggregates with platelets enables the shielding of tumor cells from shear stress and increases their metastatic potential [291].

Platelets also regulate inflammation and recruit neutrophils. In pancreatic ductal adenocarcinoma, patient samples revealed that neutrophils may assist with distant metastasis through CTC interactions [323]. In breast cancer samples, CTCs were associated with neutrophils, and these cells were characterized by a transcriptomic profile highlighting cell cycle progression, cell–cell junction, and cytokine-receptor pairs, hence expanding their metastatic potential [324]. The interaction of ICAM (Intercellular adhesion molecule)-1 between cancer cells and neutrophils enabled the intravasation of this cluster and increased their metastatic potential [325,326]. The NETs produced by activated TANs have also demonstrated a role in CTC metastasis, particularly by sequestering them by ITGβ1 expression on both TANs and CTCs [327].

#### 4.7.2. Anoikis Resistance

In addition to physical shielding from the fluid shear stress and the immune system, cancer cells must resist the cell death induced upon entering circulation. Anoikis, the Greek word for “homelessness”, is apoptosis induced due to a lack of, or inappropriate, cell adhesion to the ECM. The inability to adhere to an inappropriate or improper ECM prevents the re-adhesion of previously detached cells from native tissue to incorrect locations, serving as a protective mechanism in normal tissue homeostasis. This is characterized by an ECM that does not support normal integrin signaling, being of the wrong composition or tissue context [328]. An improper ECM or complete loss of ECM anchorage eliminates FAK signaling [329], thereby inhibiting the induction of PI3K/Akt [330] and MAPK pathways [331], reducing pro-survival signals [332], and promoting apoptosis [333]. Anoikis may also be initiated upon the temporary detachment of focal contacts during the cell migration process. Resistance to anoikis is a hallmark of cancer cells, mediated by various factors including growth factors, pH, and hypoxia [334]. The induction of EMT mediates anoikis resistance, as epithelial cells require attachment for survival, which is vital for development and tissue homeostasis. EMT circumvents this by adopting a mesenchymal motility style with elongated morphologies and cell polarity, enabling integrin engagement and the activation of RTKs, as well as activating PI3K [335].

Collagens binding their preferred DDR can result in signaling events, including FAK signaling to induce EMT and resist anoikis [31,336,337], as well as PI3K/Akt-NFκB signaling to prevent apoptosis [338]. Collagen–integrin signaling also protects against anoikis [339] through their downstream signaling pathways, including FAK, integrin-linked kinase, SFK, and MAPK [340,341,342,343,344]. Hence, a specific switch in integrins and their activation and overexpression are common mechanisms by which cancer cells prevent anoikis [345,346]. Collagens have been suggested as targets in anoikis resistance, including collagen type V in glioblastoma [347] and collagen type XIII in breast cancer [339]. In colorectal carcinoma, anoikis resistance was characterized by samples with high collagen type IV and laminin expression [348]. In agreement with this, pathway analysis has shown that anoikis-related gene signatures were enriched in collagen-containing ECM and integrin binding, PI3K/Akt signaling, and apoptotic signaling [349].

Cancer cells can inhibit apoptosis through the downstream mammalian target of rapamycin (mTOR) signaling and inhibition of caspase activation [57]. Changes, both biochemical and environmental, can activate apoptosis both intra- and extracellularly. This mechanism of apoptosis converges on pathways that result in the activation of caspases and their downstream molecular pathways, ultimately causing cell death. The interplay between the intrinsic and extrinsic pathways of apoptosis induces anoikis, as disengagement from an appropriate ECM leads to the activation of both the intrinsic pathway, involving perturbation of mitochondria, and the extrinsic pathway, involving the triggering of cell surface death receptors. Activation of the extrinsic pathway occurs through the engagement of death receptors, including Fas, TNF-receptor 1, and TNF-related apoptosis-inducing ligand receptors 1 and 2, which form the death-inducing signaling complex (DISC) [350]. DISC interacts with adaptor proteins, such as Fas-associated death domain protein, which then recruits and activates caspase-8 [350,351]. This results in cleavage and activation of executioner caspases (caspase-3) and Bid, which links the extrinsic and intrinsic pathways. Activation of the intrinsic pathway, through Bcl2-homology-3-only proteins (Bim, Bad, and Puma), promotes Bax/Bak activation directly or indirectly via Bcl-2 [352]. Reports have also indicated that the mitochondrial protein Bit1 is released into the cytoplasm upon cell disengagement from the ECM and serves as a pro-apoptotic mediator [353,354]. This results in the release of cytochrome c into the cytoplasm, inducing the formation of the apoptosome and the activation of executioner caspases. Additionally, the release of the second mitochondria-derived activator of caspases and serine proteases inhibits the inhibitors of apoptosis, thereby enhancing the effector caspases [355,356]. Both pathways converge on the initiation of the downstream proteolytic cascade to cell death, which is ultimately mediated by caspase-3 [357]. Acquiring mutations that constitutively activate anti-apoptotic pathways or loss of tumor suppressors can promote survival [342,358]. Upregulation or activation of RTKs can result in pro-survival pathways overriding the loss of integrin signaling or apoptosis-promoting pathways and suppressing anoikis [359,360,361]. Therefore, the ability to circumvent these signaling cascades, largely mediated by EMT, enables survival of CTCs in circulation.

The formation of hetero- and homo-typic microaggregates described above is another mechanism to prevent anoikis. Studies have shown that, specifically, synoikis-like behavior supports cell survival [362]. Synoikis is the formation of cell aggregates in nonadherent conditions via E-cadherin interaction between neighboring cells, and is proposed as a mechanism of anoikis resistance [363,364]. Additionally, fibronectin upregulation, resulting in cell aggregate formation, enhances anoikis resistance [365]. Studies show that the size and number of aggregates that tumor cells form correlate with their survival [366].

Anoikis resistance plays a role in several steps of metastasis. The ability to resist apoptosis without adhesion or adhesion to an improper matrix perpetuates the autocrine signaling of cytokines and growth factors, resulting in a larger tumor size. Still at the primary tumor site, as cells undergo EMT and lose cell–cell adhesion, the acquisition of anoikis resistance is necessary for the survival of these cells. Lastly, in addition to survival in circulation, the distant metastatic site serves as an improper ECM, and hence, anoikis resistance is also vital here.

#### 4.7.3. Immune Evasion in Circulation

While the primary tumor site promotes an immunosuppressive environment, upon entering circulation, these tumor cells again risk exposure to the immune system. Key immune cells with anti-tumor effects in circulation include T cells and natural killer (NK) cells. Leukocytes originate in the bone marrow and migrate to and circulate in the bloodstream and lymphatics. In addition to the physical shielding by microaggregate formation described above, antigen modulation, immune checkpoint activation, and alterations in immune cell recruitment can occur.

Secretion of CXCR (CXC chemokine receptor)-1 and CXCR2 by tumors induces NETs to prevent T cell and NK cell contact [367]. Platelets secrete TGF-β, activating immunosuppressive Treg cells [368,369]. Platelets also enable molecular mimicry, as tumor cells can become rapidly coated in platelet-derived MHC-1, preventing NK cells from killing [370]. Tumor cells themselves also inhibit the anti-tumor activity of NK cells through shedding NK group 2D ligands [371], secreting lactate dehydrogenase isoform 5 [372], downregulating DR5 [373], and secreting immunomodulatory molecules [291].

Evasion of other cells in circulation, including macrophages, has been achieved through upregulation of CD47 [374] and CD24 [375]. Immune checkpoint molecule PD-L1 expression on CTCs [376], CTMs [304], CAFs [377], and on the lymphatic vessels themselves [378] is a potent anti-immunity mechanism in circulation. Rapidly extravasating into the surrounding tissue is also beneficial for cancer cell survival, as it circumvents the immune system, anoikis signaling, and mechanical stress.

### 4.8. Extravasation

Cancer cells must extravasate into a secondary organ to complete metastasis. Extravasation occurs due to the arrest of CTCs, achieved by adhesion to the vessel’s walls with the help of supporting cells, including arrest by platelets and myeloid cells in microvessels of various organs, or occlusion if the vessel is too narrow. Both lead to the subsequent large size of the tumor that forms as proliferation continues, enabling extravasation at the distant site.

Paracellular migration appears to be the dominant mechanism of extravasation, whereby tumor cells migrate between endothelial cells through cell rearrangements and disruption of cell junctions. Therefore, active adhesion is necessary in addition to physical arrest by microvessels. Evidence for the role of integrins, cadherins, and selectins in vitro has been demonstrated [291]. For cancer cell adhesion to the vessel walls, adhesion molecule expression, including immunoglobulin superfamily member gene 3 [379], CD44 [380], MUC1 [381], CD146 [382], platelet endothelial cell adhesion molecule-1 [383], or VCAM (vascular cell adhesion molecule)-1 [384] has been upregulated. Osteonectin has been demonstrated to promote extravasation in melanoma cells, upregulating VCAM-1 [385]. ICAM-1 expression has also been shown to be induced in various cancers via tumor-derived IL-35 [386] and fibrinogen [387]. Platelet GPVI has also been shown to promote extravasation through cancer cell-derived galectin-3 [388].

Cancer cells can also promote extravasation directly through the secretion of angiopoietin-like 4, which antagonizes vascular endothelial junctions, thereby facilitating the extravasation of breast cancer cells [389]. Alternatively, the release of damage-associated molecular patterns induces the opening of the cell barrier and secondarily recruits the immune system, which may also promote extravasation. Platelets also promote extravasation through the release of dense-granule-derived adenosine triphosphate, modulating the endothelial junctions, and recruiting granulocytes to platelet–tumor aggregates [390,391]. Neutrophils can arrest CTCs through integrin signaling, and NETs may trap CTCs and facilitate adhesion to endothelium before or during extravasation [324,327,392,393,394]. TGF-β signaling has been implicated in this process [395]. The interaction of neutrophils with CTCs strengthens ICAM-1 affinity [396]. Therefore, the interaction of tumor cells with platelets and immune cells can promote interaction with endothelial cells of the vasculature, enabling early metastatic processes.

The biomechanical and biochemical cues mediated by ECM stiffness in the vasculature also play a role in tumor cell extravasation. Tumor stiffness, owing to collagen deposition, increases cancer cell extravasation and correlates with increased MMP-9 expression [397]. Collagens and their interactions with tumor cells and platelets also play a role in extravasation, mediated by HSP-47 [398]. In pancreatic ductal adenocarcinoma, collagens type I, III, and V regulate extravasation [399]. In epithelial ovarian cancer, collagen type VI knockdown resulted in reduced dissemination of metastatic cells [400]. Additionally, collagen type IV remodeling, evident by discontinuities in the vasculature, localized to breast cancer cell sites served as passageways for transmigrating cells [401]. These breaks in the BM could induce dissemination through direct contact of cancer cells with collagen type I [402]. ECM components enable redistribution of chemokine receptors on tumor cell surfaces, promoting their extravasation at specific sites [403].

### 4.9. Colonization

Once extravasated from the blood vessels at the distant organ, micrometastases are formed. EMT reverses here and is referred to as mesenchymal–epithelial transition (MET), whereby the disseminated tumor cells (DTCs) restore their epithelial phenotype and functions. These micrometastases must adapt to their new TME and reprogram the surrounding stroma to promote growth and form larger macrometastases [404]. Angiogenesis is induced again to allow for growth at this distant site by providing nutrients and oxygen. In healthy tissue, the collagen matrix density and fibril size enable physiologically routine immune surveillance; however, their modulation in the TME with overproduction of glycoproteins can elicit immune exclusion at both the primary tumor site and distant metastatic sites [57,106,405]. The ability to colonize at a secondary site is the most significant determinant of the patient’s final outcome, as the inability to metastasize successfully eliminates metastasis-associated deaths.

It is essential to recognize that not all tumors employ the same molecular strategy for metastasis, as different tumors exhibit a preference for metastasizing to specific distant sites [406,407]. There are two theories behind the organotropism of cancer: (1) the vascular connection theory and (2) the seed and soil theory. Vascular connection theory is mechanistic, owing to the pattern of blood flow to the secondary site preference. These metastatic tropisms include appropriate growth factors or ECM environment, compatible adhesion sites, and chemotaxis, all promoting the survival of the cancer cells at the distant organ. For example, IL-6-STAT3 signaling promotes metastasis to the liver [408], and caveolin-1 in breast cancer cell-derived exosomes modulates the pre-metastatic niche, promoting a favorable ECM deposition [409]. ECM core matrisome components, including fibronectin, tenascin C, osteonectin, periostin, and collagens, have all been implicated as crucial components regulating the metastatic niche in various cancers [3,409,410,411,412,413,414,415].

It has been elucidated that EZH2 mediates stemness and metastasis capacity to the bone through TGF-β/SMAD signaling, affecting collagen content and LOX activity [416,417]. Additionally, LOX is a strong determinant of bone colonization [418], as it mediates bone marrow cell recruitment that forms the premetastatic niche through crosslinked collagen type IV [419]. Weak expression of LOXL4, and subsequent increase in collagen type I and IV, is associated with poor survival in breast cancer and lung metastasis [420]. ITGα3/ITGβ1 has been shown to regulate the metastatic TME, specifically ITGα3 has a role in preventing colonization [421]. On the other hand, DDR1 signaling is necessary for cell survival, homing, and colonization of lung cancer bone metastases [422]. Inhibition of collagen type I expression in the lung prevented lung metastasis [423]. Collagen type VI derived from cancer cells or the stroma is essential for the metastatic niche of pancreatic cells [424]. In bladder cancer cells expressing collagen receptor CD167a, collagen stimulation promotes its metastasis from the primary tumor site but is also utilized to preferentially colonize airway smooth muscle cells in the lung, as they are rich producers of collagen type III [425]. In high-grade serous ovarian cancer, collagen access enables fallopian tube colonization through PTEN (Phosphatase and TENsin homolog deleted on chromosome 10) activation [422]. Hence, specific tissues are more hospitable environments for metastatic cells, while others do not support cancer growth. Even at the metastatic site, ECM remodeling can occur early in metastasis to facilitate CTC colonization and enable them to thrive in their distant organ. For example, metastatic breast cancer cells increase collagen type I density in lymph nodes [426]. Collagen type I mineralization in bone marrow facilitates breast cancer metastasis, as the increased glycocalyx thickness resists NK cell attack [427]. Similarly, deposition of collagen type I found in liver metastases of melanoma cells is owed to the upregulation of CXCR4 and CXCL12, promoting Akt/NFκB signaling, and is associated with a reduction in tumor-infiltrating lymphocytes and enabling survival of the micrometastases [428]. Remodeling of the distant metastatic niche into a fibrotic, collagen-rich environment is therefore a potent pro-metastasis tumor strategy [429].

#### Dormancy Escape

Primary tumors and macrometastases consist of the characteristic highly proliferative polyclonal tumor cells and, therefore, are typically detectable and targetable. However, during clinical latency, the tumor cells can enter a state of cell cycle arrest or tumor dormancy at distant sites [430]. In this critical stage, these cells are likely shielded from immune detection and treatment before their transformation into lethal macrometastatic lesions. Evidence suggests that cancer cells become dormant to survive in at least three different scenarios, including primary cancer dormancy, metastatic dormancy, and therapy-induced dormancy [104]. Dormancy may be established by the tumor cell as a protective mechanism against therapeutic strategies, either directly or through the hypoxic environment they cause [431,432]. In estrogen receptor α-positive (ER+) breast cancer, recurrence decades after successful initial treatment has been shown to be, in part, due to the establishment of dormancy of DTCs [433]. These cells were further characterized by decreased E-cadherin and increased ZEB1,2 expression, which is characteristic of both EMP and dormancy [433]. These dormant cells are also characterized by their immune evasion phenotype, including expression of PD-L1 [434], and immunosuppressive cytokines.

Stromal injury in the bone marrow may trigger dormancy escape through TGF-β1 signaling [435]. STAT3 is also linked to proliferation and downregulation of dormancy markers [436]; autophagy-related gene-7 promotes dormant breast cancer cell survival [437], and hypermethylation of nuclear receptor subfamily 2 group F member 1 (NR2F1) promoter is evident in dormancy models [438]. The ERK/p38 ratio and activation of the NFκB pathway have correlated with dormancy [439,440].

Matrix stiffness has been shown to modulate dormancy in various cancer types [118]. Mechanistically, waking dormant DTCs and promoting their proliferation has been achieved through a stiff ECM, resulting in the accumulation of troponin T1 [441]. In breast cancer, stiffening of the ECM regulates tumor stemness and quiescence via ITGβ1/3 receptors dependent on DDR2/STAT1/p27 signaling, and removal of this mechanical force leads to vigorous proliferation [28]. Additionally, low uPAR and ITGα5/ITGβ1 avidity reduced ERK activation, promoting dormancy in liver cancer cells [442]. Collagen type I demonstrated functions in triggering escape from dormancy and driving proliferation through ITGβ1 signaling [443]. This was confirmed as the fibrillar structure of collagen type I and its ability to engage in ITGα2/ITGβ1 signaling plays a role in growth arrest and p27 expression, imperative to dormancy activation and awakening [107]. Collagen type XVII has been suggested to play a role in dormancy maintenance and resisting chemotherapy in human colon cancer [444]. Collagen PH sustains dormancy through mitochondrial activity modulation, and its removal triggers DTC awakening, therefore highlighting the role of collagen homeostasis in tumor cell dormancy [445]. Therefore, the regulation of tumor dormancy is imperative for the successful establishment of macrometastases and contributes to delayed recurrence, and collagen deposition and modification play a large role.

### 4.10. Therapeutic Resistance and Recurrence

Collagen deposition and crosslinking can also promote tumor development at both the primary tumor and metastatic site through therapeutic resistance. It can serve as a physical shield to block access to therapeutic strategies, and at times can be induced as a result of therapeutic intervention, leading to disease recurrence. Analysis of tumor collagen characteristics following trastuzumab treatment showed increased collagen density and decreased alignment in breast cancer xenografts [446]. In ovarian high-grade serous carcinoma, fibrotic ECM heterogeneity was evident with chemotherapy, highlighting co-evolution of the matrisome, especially stiffness and ECM composition, as mediators of resistance to platinum-induced apoptosis via FAK and ITGβ1-YAP signaling [447]. Treatment of hepatocellular carcinoma cells with cistplatin reduced apoptosis when cultured on stiff compared to soft ECM, and this was associated with increased stem cell markers, including CD44, CD33, and CXCR4 [118]. In pancreatic cancer, collagen type I can differentiate responders from non-responders of neoadjuvant chemoradiotherapy [448] and allows these cells to override checkpoint arrest induced by gemcitabine through ERK signaling [449]. It has been demonstrated that a fibrotic response to therapies are able to predict survival and recurrence [450].

Collagen type IV was identified as an independent prognostic factor in pancreatic ductal adenocarcinoma, predicting prognosis and resistance to neoadjuvant chemotherapy-treated patients after surgery [451]. Similarly, in triple-negative breast cancer, 75% of patients were unresponsive to chemotherapy and were characterized by an upregulation of collagen type IV expression, which mediated invasion and metastasis through SFK and FAK signaling [452]. In a subset of urothelial carcinoma cells, collagen type IV and ITGβ1 signaling not only elevated EMT but also promoted gemcitabine resistance and recurrence [453]. Collagen type IV signaling through ITGαV was also shown to be correlated with immunotherapy resistance in advanced clear cell renal carcinoma [454].

In ovarian cancer, cistplatin resistance was mediated by collagen type XI signaling through ITGα1/ITGβ1 and DDR2 downstream Src-PI3K/Akt-NFκB signaling to induce the inhibitor of apoptosis proteins XIAP, BIRC2, and BIRC3 [455]. Similarly, collagen type II expression was a predictor of tumor recurrence in high-grade serous ovarian cancer [456]. Activation of the PI3K/Akt-NFκB signaling pathway and its induction of gemcitabine resistance were also demonstrated in high collagen type VIII pancreatic ductal adenocarcinoma, upon DDR1 and ITGβ1 activation [457].

Inhibition of collagen PH A4 sensitizes triple-negative breast cancer cells to chemotherapies docetaxel and doxorubicin through HIF-1-dependent cancer cell stemness modulation [458]. Similarly, LOX inhibition led to better drug penetration, inhibited FAK signaling, induced ROS/DNA damage and G1 arrest, and hence enhanced the chemoresponse of triple-negative breast cancer [459]. Blocking of collagen and chemokine expression through CCN family member-1 was also able to modulate gemcitabine and anti-PD-1 sensitivity in pancreatic ductal adenocarcinoma, through increased cytotoxic immune cell infiltration [460].

Collagen content and alignment were also independent predictors of recurrence in prostate cancer and breast ductal carcinoma, respectively [461,462]. Collagen type I fiber volume was shown to predict recurrence in non-small cell lung cancer [463]. Collagen type I also induces EGFR-TKI resistance in lung cancer cells with mutated EGFR through mTOR activation [464]. Collagen type I was also implicated in breast cancer chemosensitization, through DDR1 and ITGβ1—mediated MAPK signaling [465].

Modulation of the immune system by collagen signaling has also been demonstrated as a mechanism of therapeutic resistance and recurrence. High levels of fibrotic tumor components, especially collagen, were associated with recurrence and decreased intratumoral immune cell infiltration in advanced colorectal cancer patients [466]. Resistance to radiation therapy and consequential recurrence of vestibular schwannoma were correlated with high expression of four collagen family genes, collagens type V, III, IV, and XV, and were significantly associated with immune infiltration [467]. In triple-negative breast cancer, collagen type V was particularly upregulated in chemoresistant samples, and the production of IL-6 was found, but triple-negative breast cancer cells overexpressing collagen type V promoted macrophage polarization, TGF-β production, and consequently doxorubicin resistance [468]. Collagen types III and VI were also implicated in non-small cell lung cancer acquired immunotherapy resistance, serving as barriers to T cell infiltration and protection from T cell attack [469]. Collagen type XI is implicated in the obstruction of T cell infiltration and chemotherapeutic resistance, and consequently recurrence in non-small cell lung cancer [470,471]. Additionally, collagen type VI was correlated with glioma cell chemotherapy resistance, particularly through immune cell infiltration [472]. Additionally, treatment of colorectal carcinomas with rapamycin modulated depleted CAFs and degraded tumor tissue collagen, enabling infiltration of T lymphocytes into the tumor tissue [473].

Collagens can modulate cancer cell signaling, but cancer cells can also modify collagen, reinforcing the cell–collagen feedback loop that will support cancer progression and influence patient outcomes. This suggests that targeting the ECM through remodeling enzymes and their receptors may be a viable strategy to enhance tumor responses to therapy.

## 5. Collagens for Cancer Prognosis and Treatment

Protein signatures can distinguish tumors from normal tissues, primary from secondary tumors, tumor stage, and tumors from other diseases. Likewise, expressions of ECM and ECM-associated genes have demonstrated value in diagnosis, prognosis, recurrence prediction, therapeutic resistance, and as a potential therapeutic target. As described above, various collagens have been implicated as prognostic factors for cancer patients, including but not limited to recurrence, metastasis, and overall survival, and with that, have been implicated as a promising anti-cancer strategy [53] (Table 1). As these effects are context-dependent, depending on the cancer type and stage, a specific collagen type could be pro- or anti-cancer. This includes modulations of their expression, degradation, methylation, and mutation.

In addition to tissue expression of collagens described above, collagens have also been identified in the serum and have prognostic value, including serum collagen type IV for granulosa cell tumor of the ovary [514], and serum collagen types XIX and XXII in small cell lung cancer patients [562]. It is also apparent that TACSs are of equal importance in cancer prognosis. Of the five collagen parameters, width appears to be the most predictive of cancer prognosis [658]. In a comparison of TACSs of those with predicted disease recurrence vs. those with disease-free survival, patients who may benefit from adjuvant chemotherapy were successfully distinguished [581]. A collagen score, based on area, fiber number, and textural feature in advanced rectal cancer patients, also demonstrated good prognostic value [659]. An analysis of 142 collagen features by second-harmonic generation (SHG) imaging showed effective prediction of recurrence and survival in patients with colon cancer [660]. SHG microscopy has been used to quantify collagen fiber morphology and organization [49] and measure dynamic changes in the TME following treatment [446]. Collagen nomograms from multiphoton imaging have also been utilized to assess collagen signatures of the TME and correlated to various prognostic factors [661,662]. Therefore, advanced imaging, such as SHG microscopy and collagen nomograms, to quantify TACSs may serve as an early detection or treatment monitoring strategy. As some cancers more commonly present with dense, aligned collagen, like triple-negative breast cancer and pancreatic cancer, strategies that modulate the TACS, like ECM-degrading agents before or in conjunction with other therapies or CAF normalization, may benefit these cancer patients, while providing less benefit to those with a less structured ECM.

The matricryptins produced following collagen degradation that are detected in serum can also serve as prognostic biomarkers (Table 1). This largely includes various fragments of collagen types I, III, IV, including ICTP, NTx, PRO-C3, and C4M. Endostatin, a collagen type XVIII fragment, has been evaluated in the serum of several cancer types [560,638,639,640,641,642,643,644,645,646,647,648,649,650,651,652]. Enzymes that modify collagen expression, including TIMP-1, MMP-8, and MMP-2, have been detected in the serum of patients with various cancer types and have also demonstrated strong prognostic value [261,597,603,655,656,657] (Table 1). Thus, collagens can serve as biomarkers, but as they are also genetically and physically stable, they are an attractive and promising therapeutic target.

Targeting the ECM, especially collagen, for cancer therapy has been previously suggested to combat the architecture and physical blocking of immune cells and anti-cancer therapies (Table 2). Targeting collagen may be especially helpful in combination therapy, serving to enhance drug delivery and therapeutic effects. Proposed strategies include collagenase treatment [663,664,665], nitric oxide donors, collagen-degrading bacteria, and oncolytic viruses [666]. Oncolytic viruses can be engineered to express proteins such as MMPs that will degrade collagen fibers. Additionally, the engineering of motile bacteria that are proteolytic has also been discussed. Targeting specific collagens with degrading enzymes has yielded mixed results [667,668,669]. There has been a paradox observed in MMP inhibitor trials, and this could be explained by some MMPs exerting anti-cancer effects, and, therefore, the broad inhibition of all MMPs may tip the balance towards pro-cancer, resulting in the unintentional suppression of anti-tumor mechanisms [669]. This could also be attributed to collagen degradation that can create bioactive fragments that may have been established through matrix remodeling in the TME. MMPs, like collagens, may have spatiotemporal expressions, and, again, broad MMP inhibition will be ineffective. MMPs also regulate each other and demonstrate functional redundancy, so compensation mechanisms following inhibition may come into play [669]. Therefore, neutralization of collagen with small molecule inhibitors, monoclonal antibodies, inhibiting CAFs, or blocking collagen receptors may be of more benefit. Preclinical studies have confirmed the usefulness of anti-FAP. Chimeric Antigen Receptor-T cell-mediated therapy targets CAFs [666,670,671,672,673,674,675]. Additionally, targeting DDR1 and LOXL2 has been described [158,666,676,677,678,679,680,681]. Targeting of integrins has been attempted, including Cilengitide, Intetumumab, and Vitaxin, with few clinical trials and limited success. This has been suggested to be a result of tumor evolution, alteration in integrin expression and their functional redundancy, poor bioavailability, and difficult trial design [682]. It has been proposed that the targeting of both the integrins themselves and the downstream signaling may yield more success. Precision targeting of collagens has resulted in the development of collagen mimetics for both targeting [683,684] and imaging in cancer [685].

In an effort to harness the pro-tumor effects of collagens, there is, for example, a clinical trial recruiting that is administering recombinant humanized type III collagen in hopes of establishing local protection and synergistic treatment in breast cancer (NCT06725082). The induced expression of COL7A1 through gene therapy is being investigated in squamous cell carcinoma (NCT06731933).

## 6. Future Directions

There are 28 collagens, some with multiple isoforms, that differ structurally, largely in their non-collagenous domains. These collagens will therefore interact with different cells through different cognate receptors and hence activate different downstream pathways. Additionally, the available cells and receptors in the TME will vary with the stage of metastasis, largely influenced by the remodeling and inflammation as the cancer progresses. Therefore, spatiotemporal progression and tracking of collagen tumor evolution with SHG microscopy from pre-malignancy to late-stage metastasis may not only enhance our understanding but also serve roles in prognosis and guide therapeutic strategy.

Future therapies should integrate ECM-targeted interventions with conventional or immune-based treatments to improve patient outcomes. Ongoing research is still needed to decode tissue-specific ECM signatures, identify actionable ECM biomarkers, and develop ECM-based combinatorial therapeutic strategies.

The investigation into oncogenic collagen variants and fragments produced in the TME warrants further in-depth investigation. The heterogeneity of collagens and their post-translational modifications may contribute to this; however, it remains to be explored. This may result in a great therapeutic advantage, as to not target global collagen expression and its receptors, but to identify abnormal collagens and eliminate or normalize them. The lack of success in targeting MMPs, integrins, and collagens, due to both the effects on normal physiology and inhibiting anti-tumor subsets, can be circumvented by the elucidation of this phenomenon. Narrowing this gap in knowledge will enhance immunotherapy and drug delivery.

In addition to the need for spatiotemporal imaging of collagens in the TME, the conventional 2D and 3D model systems may partially explain the lack of translatability from preclinical to clinical trials. While improving, the lack of the ability to integrate the immune system, vasculature, and other ECM components hinders this.

## 7. Conclusions

Metastasis is the primary cause of cancer mortality, driven by a complex series of steps: local invasion, intravasation, survival in circulation, extravasation, and colonization. Collagen, the most abundant ECM protein, plays dual roles in suppressing and promoting tumorigenesis, serving as both the metaphorical shield and sword. It is clear from this review that the context, including cancer type and stage, plays an immense role in this.

Largely, the tumor-associated ECM remodeling creates a pro-tumorigenic and immunosuppressive microenvironment that promotes therapy resistance and disease progression. This includes ECM deposition, crosslinking, and degradation. Interestingly, matrix degradation products, including collagen-derived fragments, can serve as biomarkers for tumor progression and metastasis and as active signaling molecules that influence immune responses and epithelial–mesenchymal plasticity. Targeting ECM components, including collagens and their modifiers (such as MMPs, LOX, DDRs, and CAFs), offers a promising strategy to enhance drug delivery, reverse immune exclusion, and disrupt metastatic niche formation.

## Figures and Tables

**Table 1:** Tissue and serum expression of collagen, collagen fragments, collagen-modifying enzymes, and collagen composition as prognostic biomarkers across various cancer types.

Collagen	Function	Cancer Type
Type I	Therapeutic Response	Breast [474] Esophageal [475] Ovarian [476,477]
	Prognosis/Survival	HNC [478] Liver [479] Bladder [480] Gastric [481,482,483] CRC [481] Sarcoma [484]
	Metastasis/Invasion	Esophageal [485] Blood [398] Prostate [486] Thyroid [487] Melanoma [488] Ovarian [489] Gastric [490] CRC [491]
	Growth/Proliferation	Prostate [486] Thyroid [487] Ovarian [476]
	Recurrence	Thyroid [487]
	Immunosuppression	Melanoma [492]
Type II	Diagnosis	Bone [493,494]
	Recurrence	Ovarian [456]
Type III	Dormancy	Breast [106]
	Prognosis/Survival	HNC [495] Lung [496] Bladder [480] Prostate [497] Sarcoma [484] CRC [498]
	Metastasis/Invasion	CRC [491]
	Therapeutic Resistance	Lung [496] Ovarian [477]
Type IV	Metastasis/Invasion	Breast [499,500] Liver [501,502] Blood [503] Gastric [504] Bladder [505] Cervical [506,507,508] HNC [509] CRC [491]
	Prognosis/Survival	Breast [500,510] Gastric [482] Bladder [511,512] Pancreatic [513] HNC [509] Ovarian [514]
	Therapeutic Resistance	Breast [500]
	Metabolism	Breast [515]
Type V	Therapeutic Resistance	Lung [516] Ovarian [517]
	Metastasis/Invasion	Lung [516] Ovarian [517]
	Prognosis/Survival	Breast [518] Bladder [480] Gastric [519] Cervical [506,507,508] Ovarian [520] Glioblastoma [521]
Type VI	Metastasis/Invasion	Bladder [522] Ovarian [400,520] Pancreatic [523] RCC [48]
	Therapeutic Resistance	Bladder [524]
	Prognosis/Survival	Gastric [482] Cervical [506,507,508] Pancreatic [523] RCC [48] Glioblastoma [521]
	Recurrence	Bladder [505]
Type VII	Prognosis/Survival	Gastric [525]
Type VIII	Prognosis/Survival	Thyroid [526,527]
	Metastasis/Invasion	Thyroid [526]
Type IX	Metastasis/Invasion	Gastric [528]
Type X	Metastasis/Invasion	Breast [529,530,531] Cervical [532]
	Prognosis/Survival	Breast [531,533] Gastric [534] Pancreatic [535]
	Immune Evasion	Breast [531]
Type XI	Prognosis/Survival	Breast [536] Esophageal [537,538] CRC [481] Ovarian [520,539,540,541] Pancreatic [535,542]
	Metastasis/Invasion	Esophageal [543] Ovarian [544]
	Therapeutic Resistance	Ovarian [539,540,541]
Type XII	Metastasis/Invasion	Breast [545]
	Prognosis/Survival	Gastric [490] Pancreatic [546]
Type XIII	Anoikis Resistance	Breast [339]
	Prognosis/Survival	Thyroid [526,527] Bladder [505]
	Immune Evasion	Thyroid [527]
	Metastasis/Invasion	Breast [339] Thyroid [527]
	Recurrence	Bladder [505]
Type XIV	Progression	Melanoma [547]
Type XV	Survival/Prognosis	Liver [548]
	Metastasis/Invasion	Breast [549,550] Liver [548,551] Fibrosarcoma [552] Pancreatic [553]
	Therapeutic Resistance	Ovarian [477]
Type XVI	Growth/Proliferation	Oral [554]
	Metastasis/Invasion	Oral [555] Glioma [556,557]
Type XVII	Growth/Proliferation	Breast [558]
Type XVIII	Dormancy	CRC [559]
	Prognosis/Survival	Lung [560]
	Metastasis/Invasion	CRC [561]
Type XIX	Prognosis/Survival	Lung [562]
Type XX	Prognosis/Survival	Nasopharyngeal [563] Breast [564]
Type XXI	Progression	Gastric [565]
	Prognosis/Survival	Bone [566]
	Therapeutic Resistance	Ovarian [477]
Type XXII	Recurrence	Prostate [567]
	Prognosis/Survival	Lung [562] HNC [568] Glioblastoma [521]
	Metastasis/Invasion	Prostate [567]
Type XXIII	Prognosis/Survival	Lung [569]
Type XXIV	Prognosis/Survival	HNC [568] HCC [570,571]
Type XXV	Prognosis/Survival	CRC [572]
Type XXVI	Prognosis/Survival	Thyroid [526,527]
Type XXVII	Diagnosis	Ovarian lymphoma [573]
	Prognosis/Survival	Glioblastoma [521]
Type XXVIII	Therapeutic resistance	Breast [574]
TACS	Prognosis/Survival	Breast [575,576,577,578,579,580] Gastric [581] CRC [582]
Serum Matricryptins		
C1M	Prognosis/Survival	Ovarian [583] Pancreatic [584] Lung [585] Melanoma [586] Breast [583,587]
PINP	Prognosis/Survival	Breast with bone metastasis [588,589] Prostate [590] RCC [591] Bladder [591] Multiple myeloma [592]
PICP	Prognosis/Survival	Breast with bone metastasis [593]
ICTP	Prognosis/Survival	Lung [594,595,596,597,598] Breast [593,599,600,601,602] HNSCC [603] Ovarian [604,605,606,607] Prostate with bone metastases [608] Esophageal [609] Multiple myeloma [610,611]
NTx	Prognosis/Survival	Breast [599,601,612] NSCLC [613,614] Lung with bone metastasis [613,615] Multiple myeloma [611]
CTx	Prognosis/Survival	Advanced and metastatic solid tumors [616,617,618] CRC [619] RCC [591] Bladder [591] Multiple myeloma [592]
C3M	Prognosis/Survival	Ovarian [583] Breast [583,587] Pancreatic [584] Biliary tract [620] Metastatic melanoma [586]
PRO-C3	Prognosis/Survival	Breast [587] Pancreatic [584] Biliary tract [620,621] CRC [622] Metastatic melanoma [586,623]
PIIIP	Prognosis/Survival	CRC [624] Ovarian [625]
IIINTP	Prognosis/Survival	HNSCC [603]
PIIINP	Prognosis/Survival	Ovarian [626] Breast [627]
C4M	Prognosis/Survival	Breast [583,587] Ovarian [583] Biliary tract [621] Pancreatic [584] Metastatic melanoma [586]
C4G	Prognosis/Survival	Glioblastoma [628]
C4M12	Prognosis/Survival	Breast [583] Ovarian [583]
7S Domain (IV)	Prognosis/Survival	Breast [627] HCC [629,630,631,632]
PRO-C5	Prognosis/Survival	Pancreatic [633]
PRO-C6	Prognosis/Survival	Biliary tract [621] CRC [622]
C6A6	Prognosis/Survival	Melanoma [634]
PRO-C8	Prognosis/Survival	Biliary tract [620]
C8C	Prognosis/Survival	Breast [635] Lung [635] Colon [635] Melanoma [635] Ovarian [635] Pancreatic [635] Prostate [635]
PRO-C9	Diagnosis	Bladder [636] Breast [636] CRC [636] Gastric [636] HNC [636] Lung [636] Melanoma [636] Ovarian [636] Pancreatic [636] Kidney [636]
PRO-C11	Prognosis/Survival	Pancreatic [637]
BP180 (PRO-C17)	Prognosis/Survival	Breast [282] Ovarian [282] Bladder [282] CRC [282] Kidney [282] HNC [282]
Endostatin (XVIII)	Prognosis/Survival	Lung [560,638,639] Bladder [640,641,642] Thyroid [643] Breast [644] Acute myeloid leukemia [645,646] Nasopharyngeal [647] Cervical [648] Lymphoma [649] Endometrial [650] Gastric [651] Multiple myeloma [652]
PRO-C20	Diagnosis	Bladder Breast [653] CRC [653] HNC [653] Kidney [653] Lung [653] Melanoma [653] Ovarian [653] Pancreatic [653] Prostate [653] Gastric [653]
PRO-C22	Prognosis/Survival	Pancreatic [654]
Serum Levels of Collagen Modifying Enzymes		
MMP-8	Prognosis/Survival	CRC [261]
MMP-2	Prognosis/Survival	Melanoma [655] Gastric [656] Ovarian [657]
TIMP-1	Prognosis/Survival	Lung [597] HNC [603]

Abbrevications: CRC = colorectal cancer, HNC = head and neck cancer, MMP = matrix metalloprotease, PRO = pro-collagen, RCC = renal cell carcinoma, TIMP = tissue inhibitor of metalloproteases.

**Table 2:** Therapeutic strategies targeting ECM/Collagens.

Strategy	Experimental Evidence/Clinical Trials	Combination Potential with Immunotherapy/Chemotherapy
Enzymatic Degradation		
Collagenase	Decreased tumor volume [663] Safe [663] selective degradation of intratumoral collagen in probiotic delivery system [664] Reduced collagen in and interstitial fluid pressure of tumor [665]	Efficient and safe with or without Mitomycin [663] Synergistic anti-tumor effects with Doxorubicin in probiotic delivery system [664]
MMP Activation	Nitric oxide activation of MMPs enhanced tumor penetration and antitumor efficacy without toxicity [667] Nitric oxide released upregulated TGF-β expression, downregulated MMP-9 expression, and increased collagen production at wound site [668]	N/A
Receptor Blockade		
Anti-DDR-1 and -2	Antitumor effects [676] Reverse immune evasion [158,677]	Frequently in combination with chemotherapy, targeted therapy, and immunotherapy for synergistic effects [678,679]
Integrin inhibitors	Decreased growth and metastasis [686] Limited success in clinical trials [682].	Effective in combination [687]
Targeting CAF		
Anti-FAP	FAP-specific CAR-T cells: inhibited the growth of FAP-positive human tumor cells; promoted survival [670], and was well tolerated in clinical trials [671] FAP-targeted antibody-radionuclide conjugate: suppressed tumors in mice without side effects [672] FAP α antibody, FAP α vaccine, and modified vaccine all inhibit tumor growth and prolonged survival in mouse models [673]	High efficacy in combination with immunotherapy [674], but does not extend survival in combination with radiation [675]
ECM Remodeling Inhibitors		
LOXL-2 inhibitors (i.e., Simtuzumab)	Inhibited tumor growth and angiogenesis [680] In combination with LOX inhibitor, saw synergism and lower overall metastatic burden [680] Decreased chemotherapy-induced desmoplasia, stiffness, invasion, and metastasis; improved tumor perfusion [681]	Enhances chemotherapy [681,688,689]

Abbreviations: MMP = matrix metalloprotease; FAP = fibroblast activation protein; LOX = lysyl oxidase; CAR = Chimeric antigen receptor.

## Data Availability

Not applicable.

## Abbreviations

The following abbreviations are used in this manuscript:

ADAMs	Adamlysins of disintegrin and metalloproteases
BM	Basement membrane
CAFs	Cancer-associated fibroblasts
CTCs	Circulating tumor cells
CTM	Circulating tumor microemboli
COL	Collagen
CXCL	C-X-C motif chemokine ligand
CXCR	C-X-C motif chemokine receptor
DDR	Discoidin domain receptor
DISC	Death-inducing signaling complex
DTCs	Disseminated tumor cells
ECM	Extracellular matrix
EGF	Epidermal growth factor
EGFR	Epidermal growth factor receptor
EMP	Epithelial—mesenchymal plasticity
EMT	Epithelial—mesenchymal transition
ENDO	Endocytic receptor
EpCAM	Epithelial cell adhesion molecule
ER	Endoplasmic reticulum
ERKs	Extracellular signal-regulated kinases
FA	Focal adhesion
FACITs	Fibril-associated collagens with interrupted helices
FAK	Focal adhesion kinase
FAP	Fibroblast activating protein
FGF	Fibroblast growth factor
GP6	Glycoprotein 6
HIFs	Hypoxia-inducible factors
HSP	Heat shock protein
ICAM	Intercellular adhesion molecule
ITG	Integrin
JNK	c-Jun N-terminal kinase
KRT	Cytokertain
LAIR	Leukocyte-associated immunoglobulin-like receptor 1
LNM	Lymph node metastasis
LOX	Lysyl oxidase
LOXL	Lysyl oxidase-like
MAPK	Mitogen-activated protein kinase
mDia	Mammalian diaphanous-related formin
MHC	Major histocompatibility complex
MMP	Matrix metalloprotease
MT-MMP	Membrane-type matrix metalloproteases
NET	Neutrophil extracellular trap
NFκB	Nuclear Factor kappa light chain enhancer of activated B cells
NK	Natural killer
NR2F1	Nuclear receptor subfamily 2 group F member 1
OSCAR	Osteoclast-associated receptor
PD-1	Programmed cell death protein 1
PD-L1	Programmed cell death-ligand 1
pEMT	Partial epithelial—mesenchymal transition
PDGF	Platelet-derived growth factor
PH	Prolyl hydroxylase
PI3K/Akt	Phosphoinositide 3-kinase/Protein kinase B
ROCK	Rho-associated protein kinase
ROS	Reactive oxygen species
RTKs	Receptor tyrosine kinases
SFKs	Src family kinases
SMADs	Sma- and Mad-related proteins
STAT	Signal transducer and activator of transcription
TACS	Tumor-associated collagen signature
TAMs	Tumor-associated macrophages
TANs	Tumor-associated neutrophils
TAZ	Transcriptional co-activator with PDZ-binding motif
TGF	Transforming growth factor
TIMPs	Tissue inhibitors of metalloproteases
TME	Tumor microenvironment
TNF	Tumor necrosis factor
Tregs	Regulatory T cells
VEGF	Vascular endothelial growth factor
uPA	Pro-urokinase-type plasminogen activator
uPAR	Pro-urokinase-type plasminogen activator receptor
UPARP	Pro-urokinase-type plasminogen activator-associated protein
VCAM	Vascular cell adhesion molecule
YAP	Yes-associated protein
